# IntroUNET: identifying introgressed alleles via semantic segmentation

**DOI:** 10.1101/2023.02.07.527435

**Published:** 2023-02-07

**Authors:** Dylan D. Ray, Lex Flagel, Daniel R. Schrider

**Affiliations:** 1 Department of Genetics, University of North Carolina at Chapel Hill, Chapel Hill, NC 27599, USA; 2 Division of Data Science, Gencove Inc., New York, NY 11101, USA; 3 Department of Plant and Microbial Biology, University of Minnesota, St Paul MN, 55108, USA

## Abstract

A growing body of evidence suggests that gene flow between closely related species is a widespread phenomenon. Alleles that introgress from one species into a close relative are typically neutral or deleterious, but sometimes confer a significant fitness advantage. Given the potential relevance to speciation and adaptation, numerous methods have therefore been devised to identify regions of the genome that have experienced introgression. Recently, supervised machine learning approaches have been shown to be highly effective for detecting introgression. One especially promising approach is to treat population genetic inference as an image classification problem, and feed an image representation of a population genetic alignment as input to a deep neural network that distinguishes among evolutionary models (i.e. introgression or no introgression). However, if we wish to investigate the full extent and fitness effects of introgression, merely identifying genomic regions in a population genetic alignment that harbor introgressed loci is insufficient—ideally we would be able to infer precisely which individuals have introgressed material and at which positions in the genome. Here we adapt a deep learning algorithm for semantic segmentation, the task of correctly identifying the type of object to which each individual pixel in an image belongs, to the task of identifying introgressed alleles. Our trained neural network is thus able to infer, for each individual in a two-population alignment, which of those individual’s alleles were introgressed from the other population. We use simulated data to show that this approach is highly accurate, and that it can be readily extended to identify alleles that are introgressed from an unsampled “ghost” population, performing comparably to a supervised learning method tailored specifically to that task. Finally, we apply this method to data from *Drosophila*, showing that it is able to accurately recover introgressed haplotypes from real data. This analysis reveals that introgressed alleles are typically confined to lower frequencies within genic regions, suggestive of purifying selection, but are found at much higher frequencies in a region previously shown to be affected by adaptive introgression. Our method’s success in recovering introgressed haplotypes in challenging real-world scenarios underscores the utility of deep learning approaches for making richer evolutionary inferences from genomic data.

## 2 Introduction

Speciation events are often followed by the two nascent species coming into secondary contact. In many cases this creates the potential for hybridization, which can in turn result in alleles crossing from one species into the other [1]. There is a growing body of evidence such that post-speciation gene flow is a common occurrence [2, 1, 3, 4]. The introgression of alleles from one species to another can have a significant impact on fitness and evolution. Introgressed alleles will presumably often reduce fitness in the recipient species, because of incompatibilities between the introgressed alleles and the recipient species’ environment or genomic background [5, 6], or because the donor species may in some cases have a higher burden of deleterious alleles [7]. In rarer instances introgression may be beneficial, especially if the species have a shared selective environment, and the donor species contains alleles that that are adaptive in this environment and that the recipient species lacks (e.g. [8, 9]). For example, in humans, an *EPAS1* allele that originated in an archaic human relative (Denisovans) and that confers greater tolerance to high altitudes, is found at high frequency in Tibetans [10]. A similar observation of adaptive introgrssion at *EPAS1* was also made in Tibetan mastiffs, who may have received adaptive alleles from Tibetan gray wolves [11]. In *Anopheles* mosquitos, alleles that increase resistance to insecticides have jumped across species barriers [12]—an alarming observation that suggests that the control of these and other pests may be made even more challenging by their potential to experience adaptive introgression. These findings suggest that, while often deleterious, introgression may also present a route to more rapid adaptation in species that are able to borrow adaptive alleles from a neighboring relative.

For these reasons, there is a great deal of interest in detecting the extent of and genomic loci affected by introgression [13, 14]. A number of statistical tests have been developed to detect the presence of introgressed alleles/haplotypes. These may ask whether there is an excess of sites in the genome that appear to exhibit patterns of inheritance that depart from the known phylogenetic relationship between species [15, 16, 17] or an excess of phylogenetic trees inferred from individual loci that differ from the species tree in a manner that is best explained by introgression [18, 19, 3]. When genomic data from multiple individuals from a given population are available, statistical tests may search for loci that have unusually similar allele frequencies between the populations experiencing gene flow [20, 21, 22], or even for haplotypes that appear to be shared between these populations [23, 24, 14]; the latter approach has the potential to identify specific loci affected by introgression. Local ancestry inference methods, which typically compare a sample of potentially admixed genomes to a reference panel of potential donor populations [25], also have the potential to reveal introgressed regions [26, 27].

Although methodological advances in the search for introgressed regions are welcome, merely assessing the presence of introgression within a genomic region has its limitations. We may wish to know how much introgression has occurred in a given region: how many sites were affected, and which individuals have introgressed material at each of these sites? Note that this information would in turn yield estimates of the frequencies of introgressed alleles in the recipient population. All of this information is useful for drawing inferences about the fitness effects of gene flow between a particular pair of populations, or even at particular loci. The development of machine learning methods for population genetic inference may represent one possible means of addressing this problem. Machine learning methods have recently made significant inroads in a number of problems in population genetics, including detecting positive selection [28, 29, 30, 31, 32, 33, 34], performing demographic inference [35, 36], and estimating recombination rates [37, 38]. We previously developed a supervised machine learning method for detecting gene flow, called FILET, that dramatically increases the power to detect introgressed loci relative to methods that use a single summary statistic [39]. More recently, Durvasula et al. created a machine learning method, called ArchIE, that infers, for each individual in a sample, whether they received introgressed alleles from an unnsampled (or “ghost”) population in a given window [40]. By averaging predictions made across all sliding windows overlapping a given polymorphism, ArchIE is capable of producing an inference at every polymorphism for each individual in the alignment.

Both FILET and ArchIE make their inferences by examining vectors of population genetic summary statistics and using simulations to train a classifier to distinguish among alternative evolutionary models, an approach that has become increasingly common in recent years [41]. However, an alternative approach that could potentially be even more powerful and flexible is to skip the step of calculating summary statistics and instead train deep neural networks to examine population genetic alignments directly as their input. For example, convolutional neural networks (CNNs; [42, 43]), which are powerful tools for making predictions from various data types including images [44], can readily be adapted to population genetic alignments as these can be treated as images, with the value at any given pixel indicating which allele/genotype a given individual has at a given cite. Chan et al. recently showed that this approach can detect recombination rate hotspots with excellent accuracy [45]. Flagel et al. showed that CNNs could be trained to solve a number of population genetic problems, including detecting selective sweeps and introgressed loci, and inferring recombination rates (see also [46]), with accuracy matching or exceeding that of previous state-of-the-art methods [47]. Subsequent studies have used CNNs to perform demographic inference [48], and detecting adaptive introgression [49]. A variant of a generative adversarial network, which seeks to distinguish between real and synthetic data, has also been used to estimate demographic parameters [50]. Additional population genetic tasks that artificial neural networks have been designed for include identifying the geographic location of origin of a genome [51], mapping genetic variation data to a low-dimensional latent space [52], and inferring dispersal distances in spatial populations [53].

Although the above examples all underscore the potential of deep learning for population genetics, for the most part they simply use classification (model selection) or regression (parameter inference) to make a prediction for an entire region/genome. However, the extraordinary flexibility of deep learning architectures makes them suitable for problems that involve the production of far richer outputs. Indeed, deep learning has been used to generating artificial genomic data [54]. Another recent interesting example that treats genetic alignments as image data but produces more detailed outputs is Hamid et al’s network, which localizes selective sweeps along a chromosome in admixed populations [55]. This method works by uses an object detection framework, which seeks to identify and draw bounding boxes around objects in an image—thus, an alignment is not only classified as having a sweep, but bounds are drawn around the location of the target of selection. An even more detailed form of image processing is semantic segmentation, where the goal is to produce a prediction for each pixel in an image identifying the type of object that this pixel belongs to. This is an ideal framework for detecting introgressed alleles, as we can in principle infer, for each allele in each individual (i.e. for each pixel), whether that allele was introgressed or not. Here, we describe IntroUNET, a fully convolutional neural network that examines a two-population alignment and infers which alleles in each individual were introgressed from the other population. We evaluate IntroUNET on simulated data where we show that it is able to infer introgression with high accuracy, including in scenarios of bidirectional gene flow. We also show that IntroUNET can be easily extended to detect ghost introgression. Finally, we examine the well-known case of introgression between *Drosophila simulans* and *Drosophila sechellia* [56, 39], demonstrating that IntroUNET can accurately identify introgressed alleles/haplotypes in challenging real-world scenarios.

## 3 Methods

### 3.1 Overview of method

In this paper we explore the potential efficacy of traditional fully-convolutional neural networks (FNNs) to detect introgressed alleles in population genetic alignments, provided that the user can supply a set of training examples where the precise introgressed haplotypes, and the individuals that harbor them, are known. Typically these training examples will be simulated under a demographic model (or a set of likely demographic models) that have been estimated from the population(s) under study. FNNs were first designed to tackle image-to-image segmentation and take multi-channel images (usually with three color channels for red-green-blue (RGB) or hue-saturation-value (HSV)) as input and return an output of the same width and height with the output pixels denoting the type of object to which the pixel belongs [57]. Our approach is to treat a multi-population alignment of a genomic window as a tensor whose dimensions are *l*×*n*×*m*, where *l* is the number of populations in the sample (*l* = 2 in all experiments in this paper, although one could adapt this approach to examine *l* > 2 populations), *n* is the number of (haploid) genomes in each population, and *m* is the number of biallelic segregating sites in the genomic window. The value for each entry in the tensor is a binary indicator specifying whether the derived allele (or minor allele, in unpolarized data) is present in a given genome at a given polymorphism in a given population. On the basis of this input tensor, the FNN infers another *l*×*n*×*m* binary tensor that specifies which alleles were introgressed from the other population and which were not, thus framing the problem as image-to-image segmentation.

#### 3.1.1 Ordering individuals within the input image

Unlike the rows of pixels in an image, the ordering of rows in population genetic alignments is not typically meaningful. Thus, for the same set of population genomic sequences, there are *n*! possible image representations we could produce that all have the exact same information, meaning that the function that we would like our neural network to learn is far from isomorphic (a one-to-one mapping). One approach to deal with this problem is to use an exchangeable neural network (following [45] and [58]) where only permutation-invariant functions (column-wise means/maxima, etc.) are applied along the “genomes” axis of the input thereby ensuring that the ordering of rows has no bearing on the output. However, standard FNNs, which were inspired by the arrangement of cortical neurons in mammalian eyes [59], use 2D convolutions which rely on the hierarchical and spatially local information inherent in visual images to learn relevant “filters” or kernels, and are by definition non-exchangeable.

One way to potentially induce hierarchical information in population genetic data, while simultaneously mitigating the many-to-one mapping issue, is to sort the individual genomic windows in a manner that may be meaningful in the context of the regression model being attempted. However, there is no obvious choice of ordering that would be the most meaningful. One could imagine using the topological order specified by the genealogical trees that the alignment resulted from, but these are not known in real data and must be inferred, and in recombining genomes the tree topology and branch lengths will vary along the sequence [60]. Flagel et al. [47] addressed this problem by sorting individuals by sequence similarity by constructing a nearest neighbor graph with some distance metric, finding that this yielded an appreciable boost in accuracy relative to unsorted alignments. It is this approach that we build on here.

One way to induce a visually meaningful structure in randomly ordered N-dimensional vectors is to use seriation. Seriation, otherwise referred to as ordination, is a statistical method which seeks the best enumeration order of a set of described objects [61]. More precisely, the method seeks to order vectors in such a way that the distance between neighboring vectors in the sequence is small and thus the total summed distance over neighboring pairs is minimized. The choice of distance metrics includes any metric function defined for multi-dimensional real fields; examples include Euclidean distance and Manhattan distance for binary vectors. Such a sorting would be similar to ordering individuals by topological order along the “average” tree describing their relatedness within the focal genomic region. As a consequence, if multiple individuals had introgressed alleles, this approach would create blocks of introgression that may be more conspicuous and thus easier for the neural network to detect. We experimented with different distance metrics as described in the Results.

Seriation is known to be NP-hard, i.e. it is intractable to find exact or globally optimal solutions because as the number of vectors or destinations, *n*, grows large, the complexity grows faster than any finite polynomial function of *n* [62]. However, when the distance measure between vectors is defined to be a metric, as we do here, seriation has been found to be APX-complete or approximable-complete, i.e. there exists algorithms which can approximate the global optimum in polynomial time given some asymptotic error bound [62]. For this project we use Google’s OR-tools [63], which performs well even on large samples.

**Figure 1:**
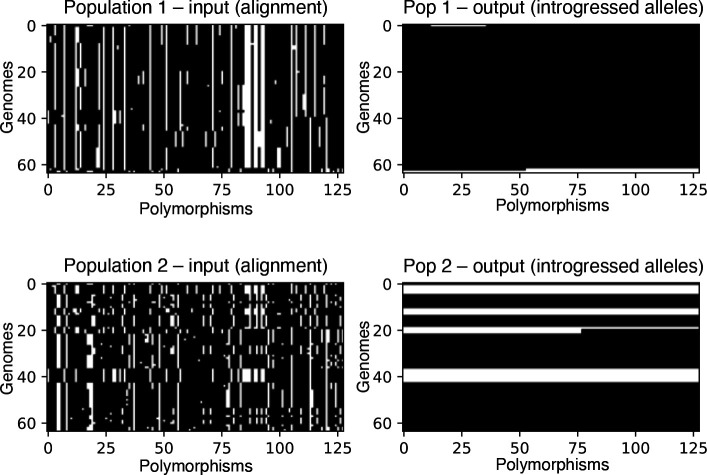
Image representation of an example input tensor (left column) and its corresponding output (right column), from a simulated scenario of bidirectional gene flow. Here, the two populations are shown as separate matrices, although they are actually part of the same input tensor (i.e. they are the two values along the “channel” dimension in the tensor). The input alignments are represented as black and white images where the ancestral allele is shown in black and the derived allele in white. The output matrices show the locations of alleles in a recipient population that were introgressed from the donor population. Thus, the white pixels in the output for population 1 show alleles that were introgressed from population 2, and the white pixels in the output for population 2 represent alleles introgressed from population 1.

Seriation orders a single population of vectors, but in our data schema there are two populations in the input tensor (dimension 1), and thus any given row in the image corresponds to two haploid individuals: one in population 1, and the other in population 2. The correspondence between these two overlying individuals may impact the ability of the neural network’s training algorithm to find meaningful 2D convolution filters. We addressed this problem by seriating one population, and then ordering the second population by performing least-cost linear matching between the seriated population and the other. Linear matching was performed between populations using the scipy Python package’s functionality which employs the Kuhn-Munkres algorithm, also known as the Hungarian method, to do so [64, 65]. Figure 1 shows, for a simulated two-population alignment, an example image representation of the input and output as specified in this section.

To summarize the intuition for the ordering and matching of populations in the image: convolutional features were designed to capture hierarchical, spatially localized information, such as stretches of similarity between individuals of different populations, and seriation and least-cost matching procedures are done to make these similarities easier to detect by the convolutional filters. The effect of these procedures on the input representation can be seen in Figure 1: note that in this example, the introgressed individuals in population 2 tend to be matched in their location along the *y*-axis with similar individuals from population 1.

#### 3.1.2 Network architecture, training, and evaluation

**Figure 2:**
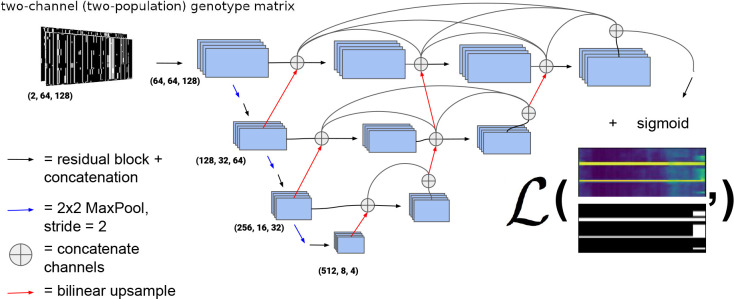
UNet++ type architecture [66] used for all the problems in this paper. The black arrows represent a residual block consisting of two convolutions where each convolution in the series is summed to the previous, and the convolution layers are concatenated before a non-linear activation (ELU) [70] is applied. The example output of the network is color scaled from 0 to 1 and represents the probability of introgression at a given allele for a given individual. The loss function (represented by the bold ℒ) is computed with the ground truth from the simulation and is the weighted binary cross entropy function (Equation 1). The weights and biases of the convolution operations are updated via gradient descent during training. The architecture we use for the problems discussed actually contains four down and up-sampling operations rather than the three portrayed here.

We chose to use a variant of the UNet++ architecture [66], a type of U-Net [67]. U-Nets are fully-convolutional neural networks that have proved capable of achieving excellent performance in semantic segmentation problems, and are so-named because of the shape of their architecture. Figure 2 shows an outline of the UNet++ architecture we chose. In the left side of the “U” a traditional CNN is trained to extract relevant features that become more and more coarse-grained due to downsampling, and then the information is passed via upsampling and concatenation to the right side of the “U” finally resulting in a binary segmentation, or binary classification for each allele in each individual (in our case as introgressed or not introgressed). In this version of a U-Net, fine-grained and coarse-grained information are synthesized together using up-sampling and skip connections. Note that in this network, upsampling steps refer to 2-dimensional bilinear upsampling, rather than transposed convolution. We implemented this network, which was used for all the problems tackled in this paper, in PyTorch [68]. For regularization, we employed both 2D InstanceNorm [69] and dropout with a rate of 0.1 in each residual block of the network. We’ve made the code publicly available and open-source on GitHub under a GNUv3 license (https://github.com/SchriderLab/introNets).

For network training, we used the Adam optimizer with default settings [71]: a learning rate of 0.001 and *β*_1_, *β*_2_ = 0.9, 0.999. For our loss function, we used the weighted binary cross-entropy function:

(1) $$ℒ(w)=\frac{1}{N}\sum_{n=1}^{N}H\left(p_{n},q_{n}\right)=-\frac{1}{N}\sum_{n=1}^{N}\left[w_{p}y_{n}\text{log}\;{\hat{y}}_{n}+\left(1-y_{n}\right)\text{log}\left(1-{\hat{y}}_{n}\right)\right]$$

where *H*(*p*_*n*_*, q*_*n*_) is the cross-entropy function of the true and predicted label distributions (*p* and *q*. respectively), the weight *w*_*p*_ is the weight for positive examples and was set to the ratio of negative examples to positive examples, *y*_*n*_ and ${\hat{y}}_{n}$ are the true and predicted labels for example pixel *n*, respectively, and *N* is the total number of example pixels in the training set. We use the weighted form of cross-entropy because the datasets examined in this paper are unbalanced in that there are far more non-introgressed alleles across individuals than introgressed alleles. Note that this data imbalance can affect evaluation metrics, and we did not construct balanced evaluation sets when computing ROC and precision-recall curves, and the former may be impacted by data imbalance. However, we also note that class-imbalance does not bias our confusion matrices obtained from these evaluation sets, and we also report unbalanced classification accuracies obtained by simply averaging the percentages along the main diagonal of the confusion matrix.

It is worth noting that our network has 4 pooling operations (and, in the second half of the network, four upsampling operations) each of which leads to a reduction (and increase in the second half of the network) in both the width and height of the input by a factor of 2. Thus, both the width and height of the input image (individual and SNP axes) must be multiples 2^4^ = 16. In the problems considered in this paper, we chose to upsample the number of individuals in the dataset to the nearest multiple of 16, and always choose SNP-window sizes which are multiples of 16. When applied to real datasets, our tool upsamples the number of individuals as necessary, which will result in multiple predictions made for some individuals, in which case our tool arbitrarily takes one of these predictions to be the final prediction for that individual. In the following sections, problem-specific details of batch size and other training specifications are given when needed.

### 3.2 Simulated introgression scenarios

#### 3.2.1 A simple simulated test case

We assessed our method’s performance on a simple simulated scenario where two subpopulations, each consisting of *N* = 500 diploid individuals, split 4*N* generations ago and later experienced a pulse of gene flow. The time of the introgression event, and the fraction of individuals introgressed, was allowed to vary uniformly from replicate to replicate. The full list of parameters and values for this model is shown in Table 1. Note that we simulated fairly small population sizes here for computational tractability, and therefore used large mutation and recombination rates, *μ* and *r*, respectively, such that 4*Nμ* = 4*Nr* = 0.02.

We simulated populations for this scenario using the evolutionary simulation software SLiM [72]. We simulate equal numbers of replicates of three scenarios: unidirectional introgression from population 1 to population 2, unidirectional introgression from population 2 to population 1, and bidirectional introgression. For each case, 10^5^ replicate 100 kb regions were simulated and a predictor and target image was created for each as described above. For the bidirectional case the target variable has two channels: one corresponding to whether a given allele was introgressed from population 1 to population 2, and the other corresponding to whether the allele was introgressed from population 2 to population 1. In the two unidirectional cases there is only a single output channel denoting whether or not there was introgression in the direction being considered. Note that we did not explicitly simulate examples where migration was disallowed, but in most simulated examples the majority of pixels in the alignment image were not affected by introgression.

**Figure 3:**
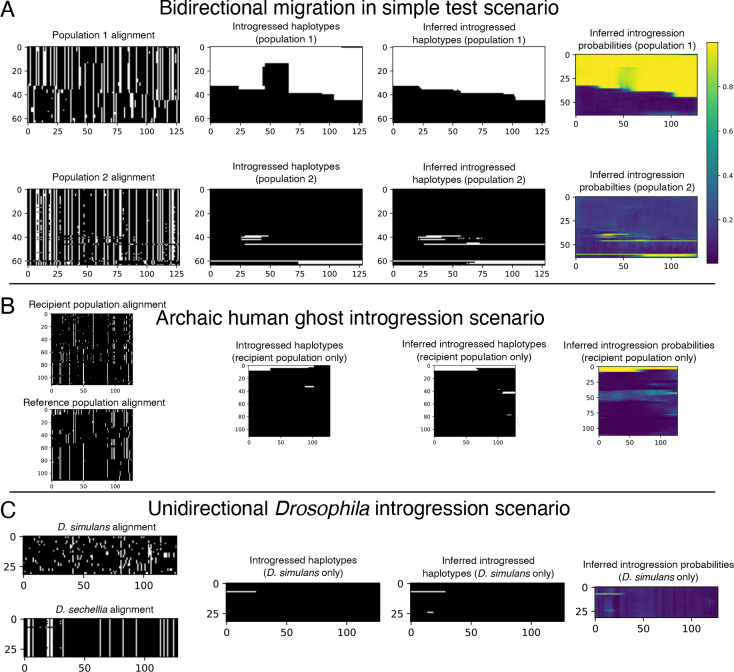
Example inputs and outputs (both true and inferred) for each of the three problems we used to assess IntroUNET’s effectiveness. (A) A simulated example of the simple test scenario of a two population split followed by recent gene flow (bidirectional, in this case). The first column shows the population genetic alignments for this example, with the two panels corresponding to the two input channels (population 1 and population 2). The second shows the true histories of introgression for this example (again, with white pixels representing introgressed alleles); note that both population 1 and population 2 have introgressed alleles. The third and fourth columns show IntroUNET’s inference on this simulation, with the former showing the most probable class (i.e. introgression or no introgression) for each individual at each polymorphism, and the latter showing the inferred probability of introgression (i.e. the raw softmax output for the introgression class). The color bar for these plots is shown in panel (A), and the scaling is the same for the panels below as well. (B) A simulated example of the archaic ghost introgression scenario. The four columns are the same as in panel (A), but here we are examining a recipient population and a reference population, with the goal of identifying introgression only in the former. Thus, our output has only one population/channel. (C) A simulated example of our *Drosophila* introgression scenario. The four columns are the same as in (A) and (B), and here we are concerned with identifying introgression from *D. simulans*to *D. sechellia*, so again our output has only one channel (i.e. introgressed alleles in *D. sechellia*).

Our initial set of 10^5^ replicates was split into training and validation sets (95% training, 5% validation), and an additional evaluation set of 1000 replicates was simulated separately but with the same parameter distribution as the training and validation sets. For this experiment we excluded from both training and testing the small number of simulation replicates that by chance had no introgressed alleles in the sampled individuals. We used a batch size of 16 examples and trained for one hundred epochs or until experiencing ten consecutive epochs with validation loss failing to achieve a new minimum (i.e. patience=10). The model with the lowest validation loss obtained was then used on the evaluation to obtain the metrics reported. An example input along with true and inferred outputs of the bidirectional scenario is shown in Figure 3A.

#### 3.2.2 Training a U-NET to detect ghost introgression

We sought to assess the effectiveness of our approach on the problem of detecting ghost introgression. Specifically, we followed the scenario of Durvasula et al. [40], where the goal is to detect introgression from an unsampled population when given data from the recipient population and an un-introgressed “reference” population. We used the same neural network architecture described above for this problem, but note that the two input channels are the recipient and reference populations, and there is only one output channel, indicating whether or not a given allele in the recipient population was introgressed from the donor ghost population. Here we train under the introgression scenario from [40], which involves a split of the ghost population, followed by the split of the reference and the recipient populations, and later followed by introgression from the ghost population to the recipient population. The parameters used to simulate this model are shown in Table 2. Simulated alignments were generated using msmodified, a version of Hudson’s coalescent simulator ms [73] modified by Durvasula et al. to track introgressed alleles. This was done using the simulation command line from the GitHub repository associated with ref. [40] (https://github.com/sriramlab/ArchIE/).

We simulated 10^6^ training/validation replicates with 5 percent of the data being randomly chosen for validation, using a window size of 50 kb and filtering windows that had no introgression. We chose to filter examples with no introgression to slightly upsample the amount of introgressed alleles as the problem is heavily imbalanced. We used the training set obtained to estimate the ratio of positive to negative alleles to weight the loss function as we describe in 1. We simulated 1000 replicates separately with a window size of 1 Mb to evaluate both methods on. For this problem, we used an input size of 2×112×192, corresponding to 2 populations (the recipient and the refernce populations), 112 individuals in each population, and 192 polymorphisms in each window examined, again with each entry being 0 (the ancestral allele) or 1 (the derived allele). The original simulation command given by [40] gave 100 individuals per sub-population, and our images of 112 individuals (the nearest multiple of 16) were created via up-sampling (i.e. arbitrarily selecting 12 individuals to duplicate in the input tensor). Our target image is simply the alleles that were introgressed from the archaic population into the recipient population represented in the form of a 192×112 binary matrix. We used a batch size of 32 when training the neural network for this problem. An example input along with true and inferred outputs of this scenario is shown in Figure 3B.

We compared the performance of our method on this task to ArchIE, the logistic model that Durvasula et al. created to solve this problem [40]. Briefly, ArchIE uses a feature vector generated for each (haploid) individual in the reference population to predict whether the individual contains introgressed alleles in a given window. ArchIE then obtains predictions for individual polymorphisms by using a sliding window and averaging the predictions for the focal individual across all sliding windows overlapping the focal site. The features used by ArchIE to make a classification for each focal individual include: the individual frequency spectrum (IFS), which is a vector showing the number of mutations present on an individual haplotype that are found at a given derived allele frequency within the recipient sample; a vector containing the Euclidean distance between the focal individual and each other individual in the recipient sample, as well as the mean, variance, skewness, and kurtosis of the distribution of these distances; the minimum distance between the focal individual and all the individuals within the reference sample, the number of singletons found in the focal individual (i.e. the derived allele is absent from both all other individuals in both samples); finally, the S* statistic [74] is included in the vector. When training ArchIE, we found that we did not achieve accuracy comparable to the original publication unless we balanced the training set. We accomplished this by randomly downsampling the number of training vectors in the non-introgressed class until the total number of vectors in both class was equal (resulting in a total training set size of ~2.6 million vectors combined across both classes). Note that no balancing was done when training IntroUNET.

For the sake of a fair comparison, the evaluation set of 1000 was kept the same between the two methods. We note that, unlike ArchIE, IntroUNET’s window size and stride are specified in polymporphisms rather than base-pairs and were chosen to be 192 and 16 polymorphisms respectively; when averaging predictions across windows, ArchIE used a window size of 50 kb and a step size of 10 kb. We also used a Gaussian window to weight predictions close the edges of the “image” smaller when averaging. This choice was made to mitigate potentially poor predictions close the edges which is known to be an issue for architectures that employ 2-d convolution due to the necessary padding operation that takes place in this part of the input tensor.

#### 3.2.3 Application to real data: finding introgressed regions in *D. simulans*and *D. sechellia*

To assess our method’s practical utility, we applied it do the dataset of 20 *D. simulans* inbred lines [75] and 14 wild-caught *D. sechellia* genomes (i.e. 7 phased diploids) previously examined in [39]. First, we obtained genotypes, phased haplotypes, and trained our method from simulated data in a manner similar to that described in [39]. We again used *∂*a*∂*i [76] to estimate the parameters of a two-population isolation-with-migration demographic model allowing for exponential population size change following the population split. This was done after mapping reads, calling variants, and phasing haplotypes (via shapeit2 [77]) in the same manner as described previously [39]. In this instance, we mapped reads to FlyBase’s [78] release 2.02 of the *D. simulans* reference genome [79] using BWA version 0.7.15 [80].

When running *∂*a*∂*i, we used the same optimization procedure as described previously [39], and once again calculated the SFS only using intergenic polymorphisms located at least 5 kb away from the nearest protein-coding gene. In our previous analysis, we had accounted for uncertainty in our estimation of demographic parameters by drawing each parameter from an arbitrarily chosen uniform distribution centered around the parameter point estimate [39]. For this study, we instead ran *∂*a*∂*i in a bootstrapped fashion by selecting with replacement which of the contiguous intergenic regions at least 5 kb from genes would be included (i.e. the allele frequencies at polymorphisms in these regions would be included in the joint-SFS for the bootstrap replicate). This was repeated 100 times, and for each bootstrap replicate, demographic parameter estimates were obtained as described previously [39].

In Supplementary Table 1, we list the demographic parameters and their estimated log-likelihood for each bootstrap replicate run of *∂*a*∂*i. For each of these parameter sets we generated 5000 simulation replicates of 0.1 Mb regions using msmodified, resulting in 215,000 replicates total. Note that although we did allow *∂*a*∂*i to infer continuous migration rates, we did not include these in our training/test simulations. Instead, we used msmove to cause a pulse-migration event from *D. simulans* to *D. sechellia* to occur in each replicate, at a time drawn uniformly from between 0 and 0.25×*N*_*a*_ generations ago, where *N*_*a*_ is the ancestral population size. The probability that any given individual in the *D. sechellia* population descended from a migrant was drawn uniformly from 0 and 1.0. We again limited the U-Net training and validation set to only those windows which contained introgressed alleles, which left 188,644 replicates total to be split into training and validation, using a random five percent of the data for validation. A separately simulated test set was generated in the same fashion, consisting of 1000 replicates containing introgressed alleles; this set was used to produce evaluation metrics reported in the Results. We chose to using label smoothing in addition to the other forms of regularization present i.e. batch normalization and dropout. Label smoothing randomly introduces uncertainty in the labels of batches via:

$$y*=y(1-\epsilon )+\frac{\epsilon }{2},$$

whereϵ~U(0,α)

Thus, true labels of 0 are perturbed up and labels of 1 are perturbed down via a randomly uniform distribution with a max of *α* which can be thought of as the strength of smoothing. For this problem, we set *α* = 0.01. We found this extra regularization was helpful to training in some problems.

During the process of training and evaluating this version of IntroUNET, we also experimented with posterior probability reclibration via Platt scaling [81], which we found to produce better calibrated estimates of the probability of introgression than the raw output from IntroUNET. The recalibrator was trained via gradient descent for 25 epochs on the unweighted cross entropy loss calculated on the validation set. This was accomplished via PyTorch and the code to do so is also included in the IntroUNET GitHub repository. An example input along with true and inferred outputs of the *Drosophila* simulated scenario is shown in Figure 3C.

As described in the Results below, the *Drosophila* version of our UNET performed best when some introgression was present in the input alignment. We therefore focused our analysis on regions that we previously found to contain introgression in the direction of *D. simulans* to *D. sechellia* (data obtained from https://github.com/kr-colab/FILET/blob/master/simSechResults/intro_regions_sim_to_sech_clustered_flybase2.02.bed), and examined our results with respect to version 2.02 of the FlyBase annotation [78] for the *D. simulans* genome. We note that this iteration of the introUNET occasionally produced false positive predictions where all of the *D. sechellia* genomes were inferred to be introgressed. Thus, to reduce the false positive rate in our analysis of the real dataset, we ignored sites that were predicted to be introgressed in more than half of our *D. sechellia* genomes. At sites that were inferred to have experienced introgression, we recorded the the fraction of *D. sechellia* individuals inferred by IntroUNET to have experienced introgressed at that site, and used this as our estimate of the frequency of introgressed haplotypes at that site.

## 4 Results

In the following section we evaluate the performance of IntroUNET on simulated data on three different scenarios (see Figure 3 for example inputs/outputs for each). The third of these scenarios is the case of introgression between *D. simulans* and *D. sechellia* [56] for which we also have a two-population sample [39] that we then use to demonstrate method’s performance on real data. We then examine two practical considerations for our method: the effect sorting of individuals within an alignment, and IntroUNET’s computational cost, respectively.

### 4.1 IntroUNET accurately identifies introgressed alleles in a simulated dataset

**Figure 4:**
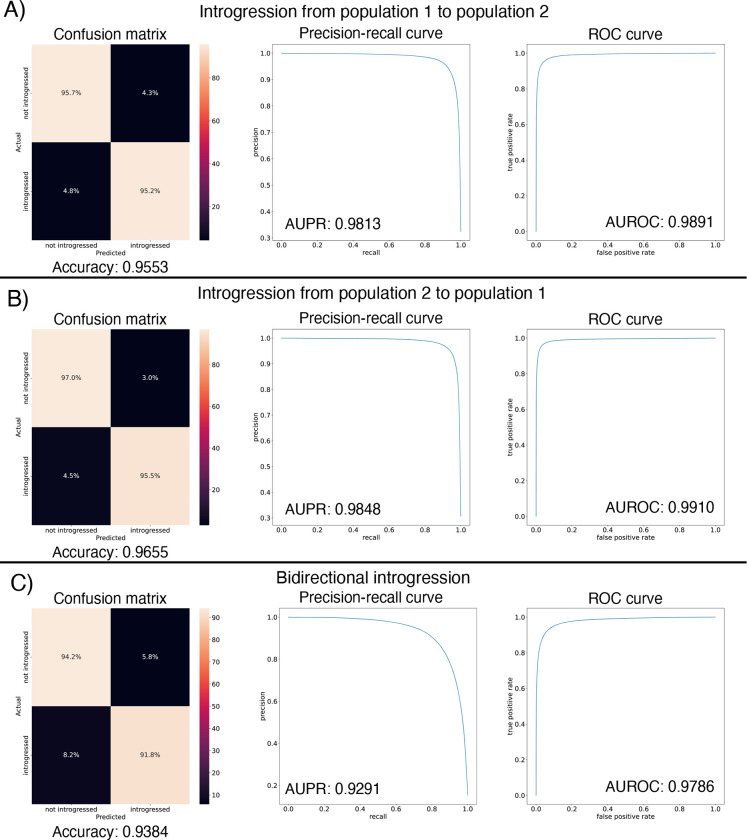
Accuracy of IntroUNET on the simple introgression scenario. (A) Confusion matrix, precision-recall curve, and ROC curve showing IntroUNET’s accuracy when trained to detect introgression in the direction of population 1 to population 2 and tested on data with introgression in this same direction. (B) Same as (A), but for introgression from population 2 to population 1. (C) Same as (A) and (B), but for bidirectional introgresion. Note that all of these metrics evaluate IntroUNET’s ability to accurately identify individual alleles (i.e. a prediction is made for each pixel in each input image in the test set, and the accuracy of this prediction is evaluated).

After designing our IntroUNET as described in the Methods, we sought to assess its effectiveness on simulated data. We began with a simple two-population model with constant population sizes, a split time of 4*N* generations ago, and introgression events occurring at times ranging between 0 and *N* generations ago (see Methods and Table 1 for more detail and Figure 3A for example input and output for this problem). We evaluated IntroUNET’s accuracy under three scenarios: introgression from population 1 to population 2, from population 2 to 1, and bidirectional introgression between both populations. We find that accuracy is very high in both unidirectional cases (e.g. area under ROC curve, or AUC, ~0.99, and area under precision-recall curve, or AUPR, ~0.98; with ROC curves, precision-recall curves, and confusion matrices shown in Figure 4A,B). Accuracy is slightly reduced in the bidirectional case (AUC ~ 0.98 and AUPR ~ 0.93; Figure 4C), which may be expected as this is a more difficult problem because individuals in either population may trace their ancestry to the other, perhaps making inter-population comparisons for the UNET more difficult. In Supplementary Figure 1, we see several randomly chosen input alignments, along with the true and predicted introgressed haplotypes. These results illustrate IntroUNET’s ability to recover introgressed haplotypes with high accuracy in this simulated scenario.

### 4.2 Reference-free inference of archaic local ancestry

Having demonstrated the efficacy of the IntroUNET on a simple scenario of introgression between two sampled populations, we next sought to investigate its performance and versatility by addressing a more challenging problem: detecting introgression from an unsampled, or “ghost”, population. A recent paper from Durvasula et al. presented a novel method for identifying regions of a genome that are introgressed from an archaic ghost population using two population genomic samples: a sample from a population that received genetic material via introgression from the ghost population, and a reference sample from a population not thought to have experienced significant introgression from the ghost population [40]. Such an approach can be used to identify alleles in the human genome that trace their ancestry to archaic human species such as Neanderthals or Denisovans. Again, we trained our and tested our method using simulations, this time generated under the model used by Durvasula et al. [40], as described in the Methods. The two main differences between this network and that described in the previous section is that the two channels of our network’s input correspond to the recipient and reference populations, respectively, and the output has only a single channel denoting whether a given allele was introgressed from the ghost population to the recipient or not (see Figure 3B for example input and output). For this problem, we also compared IntroUNET’s performance to that of ArchIE, the logistic model created by Durvasula which uses a vector of statistics to infer whether a given individual contains introgressed alleles from the ghost population. Note that by averaging predictions across sliding windows, ArchIE can be used to obtain segmentations similar to those produced by IntroUNET (see Methods and [40]).

**Figure 5:**
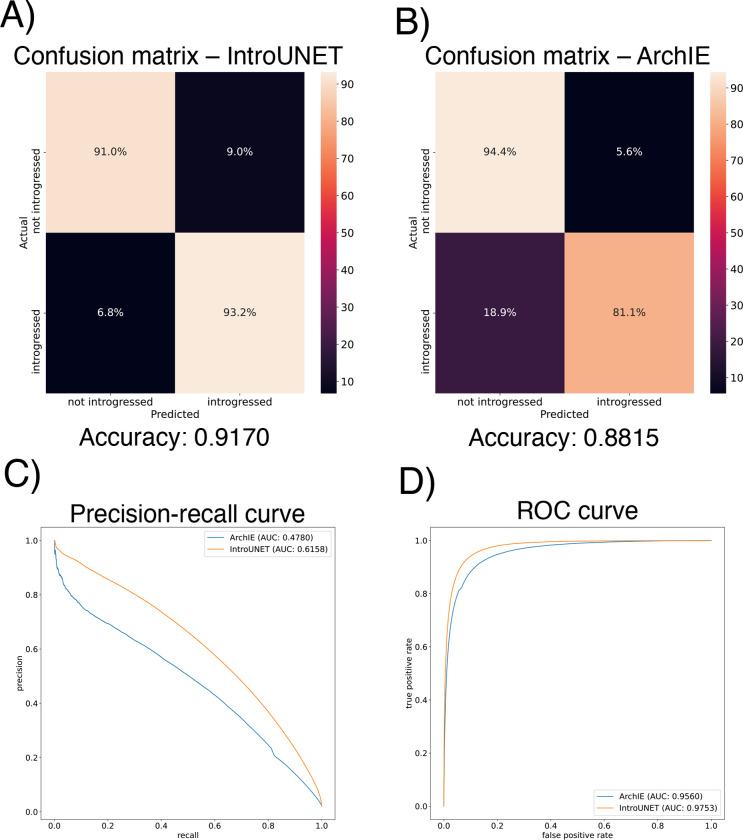
Accuracy of IntroUNET and ArchIE on the archaic ghost introgression scenario. (A) Confusion matrix, precision-recall curve, and ROC curve showing IntroUNET’s accuracy when trained to detect introgression from a ghost population to a recipient population when given population genetic data from the recipient population and a closely related reference population. (B) Performance of the ArchIE method [40] on the same scenario.

We find that IntroUNET and ArchIE perform similarly on this problem. The metrics reported in Figure 5 suggest that IntroUNET has slightly better accuracy than ArchIE on this task, and this is supported by an examination of the ROC and precision-recall curves also shown in Figure 5. However, we note that the confusion matrices shown in Figure 5 reveal a higher false positive rate for IntroUNET than ArchIE, with a higher false-negative rate for ArchIE. Given that in cases of rare introgression, false positive rates will be of greater concern, this result suggests that a more stringent classification threshold may be necessary in this scenario—an option that users can easily adjust from the IntroUNET command line. Finally, we note that IntroUNET achieved accuracy metrics substantially lower than for the scenario tested in the previous section, where data from both the recipient and donor populations are available, underscoring that this archaic ghost introgression scenario is a more difficult task. Nonetheless, IntroUNET’s relative effectiveness on this problem demonstrates that IntroUNET is a versatile framework for detecting introgression, as it can readily be adapted to very different scenarios without the need to adopt a different set of specialized statistics for the task at hand. Example segmentations produced by IntroUNET and ArchIE are shown in Supplementary Figure 2.

### 4.3 IntroUNET accurately detects introgressed haplotypes between *D. simulans* and *D. sechellia*

We had previously developed a machine learning method, called FILET, for detecting introgressed loci and applied it do data from *D. simulans* and *D. sechellia*, training it on a demographic model that we estimated from these two species [39]. While this effort revealed genomic regions that were introgressed, predominantly in the direction of *D. simulans* to *D. sechellia*, FILET can only predict whether a given window is introgressed, and cannot reveal the boundaries of introgressed haplotypes and the individuals having them. Thus, we sought to revisit this dataset as both a real-world proof-of-concept for our new method, and also to characterize patterns of introgression between these two species in greater detail.

Because the joint demographic history of these two species of *Drosophila* is considerably more complex than those of the test cases considered above, we first sought to evaluate IntroUNET’s performance on data simulated under a demographic model estimated from these data. As we had previously [39], we modeled the demographic history of *D. simulans* and *D. sechellia* using a two-population isolation-with-migration model, allowing for exponential population size change in the two daughter populations, but having a constant ancestral population. To account for uncertainty in the estimated parameters of this model, we ran 100 bootstrap replicates using *∂*a*∂*i as described in the Methods, filtered replicates with low likelihoods, and simulated an equal number of examples under each of the remaining 43 inferred demographic models. As before, we omitted continuous migration from these simulations, only including single pulse migration events in simulated examples with introgression in order to control the timing of migration and track introgressed alleles (following [39]). Because we previously found that gene flow between these two species was primarily in the direction of *D. simulans* to *D. sechellia*, we modified our IntroUNET to detect introgression in this direction only (i.e. each allele in each chromosome was classified either as not introgressed, or introgressed in the direction of *D. simulans* to *D. sechellia*). We then used these simulations to train and evaluate IntroUNET as described in the Methods (see Figure 3C for example input and output).

**Figure 6:**
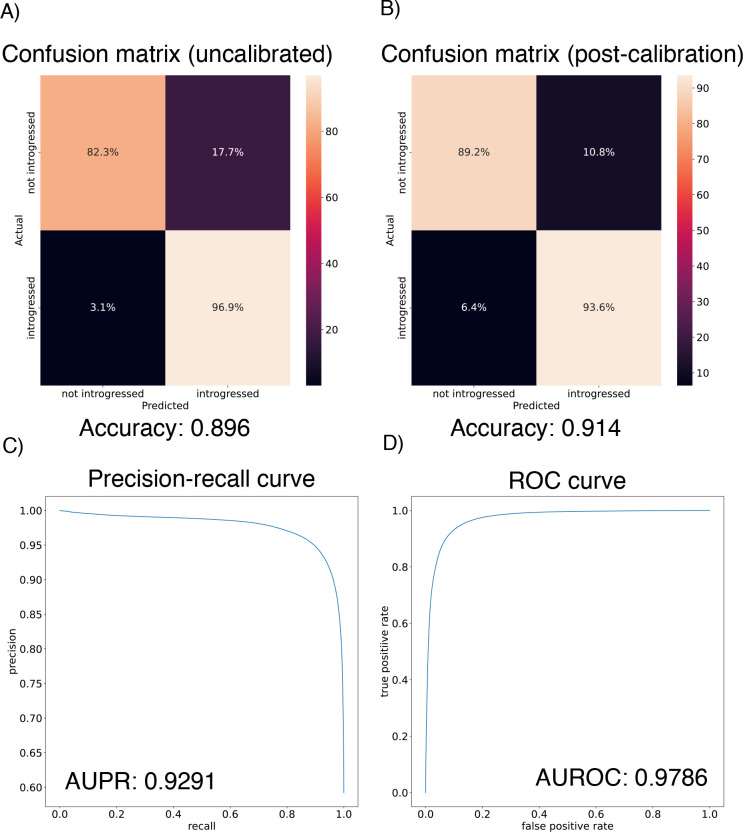
Accuracy of IntroUNET on the *Drosophila* introgression scenario. (A) Confusion matrix for the uncalibrated IntroUNET when applied to test data simulated under the *Drosophila* scenario as specified in the Methods. (B) Confusion matrix for the reclibrated IntroUNET. (C) and (D) show the Precision-recall and ROC curves for the *Drosophila* IntroUNET; note that these curves are not affected by recalibration.

We found that the IntroUNET was able to identify introgressed alleles in this simulated scenario, although the accuracy was not quite as high as observed for our simple test case described above (~90% in Figure 6 versus ~95% for the unidirectional cases in the simple scenario in Figure 4). We did observe that segmentations tended to be quite accurate within simulated regions that had experienced introgression (see Supplementary Figure 3 for examples), but a substantial number of false positives were produced in simulated regions that had no introgression. We therefore limited our analysis to regions of the genome that we previously showed to be affected by introgression (see Methods and [39]). We also note that accuracy improved from 89.6% to 91.4% after recalibrating IntroUNET’s probability estimates using Platt scaling [81]; an examination of the calibration curve (Supplementary Figure 4A) and the confusion matrices before and after recalibration (Figure 6A,B) reveal that the uncalibrated version of IntroUNET was overestimating the probability of introgression, and that recalibration corrected this (Supplementary Figure 4B). We therefore used the recalibrated version of IntroUNET in our analysis below.

**Figure 7:**
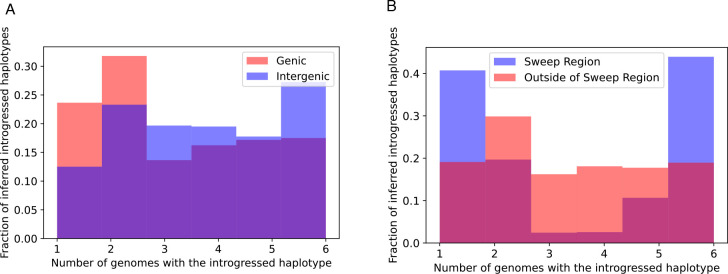
The distributions of predicted frequencies of introgressed haplotypes in A) genic (red) and intergenic (blue) regions across the genome and B) the sweep region on chr3R (blue) and other regions of the genome (red).

We reexamined the 246 10 kb windows that we previously found to be introgressed from *D. simulans* to *D. sechellia*, using IntroUNET to identify introgressed haplotypes in these regions. These windows contained an average of 2086.5 SNPs, of which 705.3 (33.4%) on average were inferred to be in at least one introgressed block (Methods); we refer to these as introSNPs. At these introSNPs, an average of 3.3 *D. sechellia* samples were inferred to have an introgressed haplotype (Methods). We next asked whether the frequencies of introgressed haplotypes differed between genic and intergenic regions of the genome. We found that introgressed haplotypes were typically found at lower frequency in genic than intergenic regions (3.2 vs. 3.7 genomes inferred to be introgressed at the average introSNP in genic and introgenic regions, respectively), consistent with the action of purifying selection against introgressed alleles. The estimated distributions of the frequency of introgressed haplotypes in genic and intergenic regions are shown in Figure 7a.

Although the above results are consistent with the notion that introgression is often deleterious, a region on the right arm of chromosome 3 (chr3R:4539900–4769900) was previously shown to have an especially large block of introgressed alleles [39] with at least one of these introgressed alleles experiencing strong positive selection within the *D. sechellia* population [82]. If this were the case, we would expect introgressed alleles in this region to be at especially high frequency, as neutral or even slightly deleterious introgressed alleles would have hitchhiked to higher frequency along with the sweeping allele(s). We find that this is indeed the case, with the average introgressed haplotype found in 3.7 individuals within this region, versus an average of 3.2 outside of this region (Figure 7b). We show IntroUNET’s predictions for three example windows within this region as well as three randomly selected introgressed windows that appear to lie outside of the region affected by adaptive introgression Supplementary Figure 5. The fact that IntroUNET infers the presence of high-frequency introgressed blocks within a region affected by adaptive introgression, but lower frequency of introgressed alleles in coding regions elsewhere in the genome, is consistent with biological expectations and implies that IntroUNET is able to make accurate inferences on real as well as simulated data.

### 4.4 Sorting via seriation improves introUNET’s accuracy

To gain some insight into what effect the decision to sort rows of the input alignment and the choice of sorting metric (i.e. the measure of distance between two sequences) might have on the ability of an FNN to detect introgressed haplotypes, we trained our architecture on the simulated *Drosophila* and archaic ghost-introgression datasets both without sorting and after seriating with various choices of metric. For the set of metrics, we used many common distance metrics used to calculate distances between vectors: L1 or cityblock distance, Pearson’s correlation coefficient, the cosine distance, the Dice coefficient, or Euclidean distance, standardized Euclidean distance, and the Russel-Rao metric. In figure Supplementary Figure 6 we show the training and validation loss obtained when training repeatedly on the same dataset but using different distant metrics or without seriating at all. We find that sorting and the choice of distance metric have a sizeable effect on the ability of our neural network’s final accuracy as measured by validation loss. In particular, the Dice coefficient metric performed the best, with cosine, city block, and Euclidean distances also performing well. Using standardized Euclidean distance or omitting the sorting step entirely both produced notably worse performance than sorting with the other metrics. These results underscore the importance of sorting data and choosing an appropriate distance metric (e.g. the Dice coefficient) when using IntroUNET.

We did not investigate whether the choice of which population is first sorted and which population is linear-sum-assigned to the former, or whether the choice of distance metric used in the linear-sum-assignment step has any effect on the efficacy of our architecture for the problems discussed here. For each problem, we chose the population that seemed to have the most diversity to be seriated and linear-sum-assigned the other to it.

### 4.5 Computational cost and implementation

Here we examine the computational cost of training and applying IntroUNET. While the simulation of synthetic alignments comes at low cost, both the training algorithm and the application of seriation to the individuals of one or more populations are more costly. We address these in turn below.

First, we compared the training times for IntroUNET and ArchIE. We trained ArchIE via the R code provided by [40] on a AMD EPYC 7413 CPU clocked at 3 GHz and IntroUNET method via an NVIDIA RTX 3090 GPU. ArchIE took roughly 7 minutes to train but large amounts of CPU RAM (> 32 Gb) as the method requires the entire dataset be made available to R. Our method took ~9.65 hours to train on this problem until convergence.

Next, we examined the computational speed of seriation, and compared this to the feature vector calculations required by ArchIE. Although the seriation problem is NP-hard, computationally tractable algorithms for approximately solving this problem do exist, and it is this approach that we take here (Methods). We found that for both the *Drosophila* scenario (*n* = 32) and the archaic introgression scenario given by [40] (*n* = 112) it took an average of 1.002 seconds to seriate one of the populations in a 128-segregating site alignment. This was calculated over 100 simulation replicates on an Intel Core i9–9900K CPU @ 3.60GHz. For comparison, it took an average of 0.08976 and 0.01701 seconds to simulate one replicate under the *Drosophila* and archaic introgression models [40] respectively, although other scenarios and simulators may require more computation. It is worth noting that the difference in sample sizes in the two examples we benchmarked (112 vs 32) did not increase average computation time (within one millisecond). We also note that the seriation routine that we used greatly outpaced the average computation time for calculating the features used by ArchIE for detecting introgression [40]—the latter took an average of 5.715 seconds to compute for each window simulated, although we took these functions directly from the repository provided and did not attempt to optimize them. Thus, although IntroUNET formats its input much faster than ArchIE, we note that for both of these methods this step can be accelerated dramatically by formatting subsets of the training set in parallel on a high-performance computing cluster, and that the routines we package with the repository to do so use MPI to accomplish this.

In order to seriate an alignment, one must define a distance metric and compute the pairwise distance matrix for all pairs of sequences in the population sample to be sorted. If we have *n* elements, each a *p*-dimensional vector then we have a complexity of *n*(*n* − 1)*/*2 multiplied by the complexity of computing the distance metric itself, which for many common metrics is simply a linear function of *p*. For instance, the computational complexity of calculating the Euclidean distance matrix is simply $\frac{3pn(n-1)}{2}$. As described in the Methods, we used the Kuhn-Munkres algorithm to perform linear matching between the two population samples in each input. Although this algorithm has a complexity of $𝒪\left(n^{4}\right)$, it takes on the order of a millisecond to terminate for the relatively small sample sizes considered in this paper when benchmarked on the same CPU mentioned above.

In short, although our method requires more training time than ArchIE, the computational cost is reasonable: when the simulation, formatting, and training steps are considered together, the entire training process can be completed on a compute cluster within one day, provided GPU resources as available.

## 5 Discussion

It has been noted that at least 10% of species experience hybridization, creating the potential for introgression [83]. Thus, in some species a sizeable fraction of the genome may be affected by cross-species introgression, and a number of methods have therefore been developed to detect introgressed loci (e.g. [24, 14, 39]). We have created a tool, called IntroUNET, that adapts a powerful deep learning method for semantic segmentation to the task of detecting alleles that have introgressed from one population to another by examining patterns of variation within an alignment consisting of samples from two populations undergoing recent gene flow with one another. We showed that IntroUNET can accurately recover introgressed haplotypes, and which individuals harbor them, in simulated data. With minimal adjustment, our method can be adapted to detect archaic “ghost” introgression by examining a two-population alignment consisting of the recipient population, and a reference population experiencing comparatively little to no gene flow. On this task, IntroUNET performs at least as well as ArchIE, a machine learning method that uses a set of features engineered for this specific task. This relatively straightforward modification to successfully attack a different introgression-detection task demonstrates the flexibility of IntroUNET, and deep learning approaches in general [47].

We were also able to apply IntroUNET to a *Drosophila* data set that we examined previously [39]. This data set consists *D. simulans*samples from mainland Africa, and *D. sechellia*samples from the Seychelles, an island nation where *D. simulans*is also present and where hybridization between the two species is known to occur [84]. It had previously been shown that there was substantial introgression between these species [56], and we had found that this gene flow was predominantly in the direction of *D. simulans*to *D. sechellia*[39]. Detecting introgression is somewhat more challenging in this data set than in the simple two-population scenario that we examined initially, most likely because of the relatively recent split time between these two species [39], resulting in much-reduced levels of diversity in this species. Nonetheless, IntroUNET performed quite well on data simulated under this demographic scenario, and when applied to the real dataset it also revealed two key patterns that were consistent with expectations, underscording IntroUNET’s practical utility. First, we observed lower frequencies of introgressed material in genic versus intergenic regions, consistent with the notion that introgression is often deleterious [6]. Second, IntroUNET predicted much higher frequencies of introgressed alleles within a region of the 3R arm that was previously shown to be affected by adaptive introgression [82], as expected under a scenario where the hitchhiking effect will cause neutral introgressed alleles that are linked to the selected allele to hitchhike to higher frequencies [85, 86]. This suggests that IntroUNET correctly identifies introgressed haplotypes even if one of the core assumptions of our training process—selective neutrality—is violated.

Although IntroUNET performed well on our *Drosophila* dataset overall, our analysis did reveal two limitations that future advances may be able to address. First, given that detecting introgression in the direction of *D. simulans*to *D. sechellia*is especially challenging (see [39]), we found that it was necessary to limit our analysis to regions that showed strong evidence of introgression. This is because, on our simulated test data for this scenario, IntroUNET infers some individuals/alleles as introgressed even in windows where no introgression is present. However, in regions where introgressed alleles are present, IntroUNET was often able to detect them accurately (Supplementary Figure 3). Second, we also observed that, on data simulated under our *Drosophila* demographic model but experiencing no selection, IntroUNET occasionally produced blocks of sites where nearly every individual was predicted (incorrectly) to have introgressed alleles. Given that we were primarily concerned with regions where only a subset of genomes had introgressed alleles, as manual examination of introgressed loci from Schrider et al. [39] had revealed that introgressed haplotypes typically appeared to be present at lower frequencies, this issue was addressed by simply filtering out all sites where the introgressed allele was predicted to be present in at least half of our *D. sechellia*sample. Another observation we made during our analysis of the *Drosophila* introgression scenario is that IntroUNET’s raw sigmoid outputs do not give well-calibrated estimates of the probability of introgression. We were able to resolve this via Platt recalibration, which produced far better calibrated probability estimates (Supplementary Figure 4). We therefore recommend this recalibration step for any analyses that hinge on the accuracy of the introgression probability estimates produced by IntroUNET, and we have incorporated this functionality into the IntroUNET software.

We demonstrated that a semantic segmentation framework can be successfully adapted to solve population genetic problems. Our method, IntroUNET, uses a variant of the UNET architecture [67], called UNET++ [66], to accurately identify introgressed alleles under a user-specified demographic history (specified during the simulation of training data) and sampling scheme. In addition to its impressive accuracy and flexibility, IntroUNET is computationally efficient, requiring on the order of a day or less for the entire training process when a high-performance compute cluster is available (to accelerate data simulation and sorting), with very rapid downstream prediction as well. Indeed, IntroUNET can feasibly be trained and run on a single consumer grade computer. However, we note that if experimentation is needed to identify the optimal neural network architecture and hyperparameters, then a cluster with multiple GPU compute nodes may be needed to make the task time-feasible. We also note that our method uses a heuristic to deal with the fact that the ordering of individuals in a population genomic alignment is generally not meaningful, and there are therefore a large number of possible image representations of a single alignment: sorting via seriation. Although this approach is relatively fast and improves classification accuracy, it is not guaranteed to produce the optimal ordering of individuals for the detection of introgressed haplotypes. Future work may obviate the need for sorting altogether by using more efficient methods that may not require sorting, such as permutation invariant neural networks [45], or Graph Neural Networks (reviewed in [87]) based on inferred tree sequences [88, 89].

## Supplementary Material

Supplement 1

## Figures and Tables

**Table 1:** Parameters of the simple simulated test case. We begin with a single population of size *N* which is allowed to “burn in” for 20*N* generations so that the populations reach, or at least approach, equilibrium. Then, a split occurs *t_S_* generations ago. After some amount of time of complete isolation, which follows the described uniform distribution, a pulse migration event occurs with individuals migrating with a probability also drawn from a uniform distribution. This migration event can occur in either direction or in both directions, and both unidirectional and bidirectional introgression is examined the Results.

Parameter	Value
Simulated chromosome size, *L*	100 kb
Sub-population size, *N*	500
Burn-in time	20*N*
Split time coefficient, *α*	4
Split time ago, *t*_*S*_	*αN*
Migration time (gens ago)	*U*(0, 0.25*t_S_*)
Migration probability	*U*(0.1, 0.5)
Mutation rate	1.0 × 10^−5^
Recombination rate	1.0 × 10^−5^
Sample size, population 1, *n*_1_	32
Sample size, population 2, *n*_2_	32

**Table 2:** Parameters of the ghost-introgression demographic model, reproduced from Table 1 from Durvasula et al. [40]. Note that our simulations used the command from Durvasula et al.’s GitHub repository (https://github.com/sriramlab/ArchIE/, which also contains a brief bottleneck experienced by the ghost population.)

Parameter	Value
Reference population size, *N*_1_	500
Target population size, *N*_2_	500
Archaic population size, *N*_*a*_	500
Burn time / archaic split time	20N
Split time coefficient, *α*	0.25
Split time ago, *t*_*S*_	*αN*
Migration time coefficient, *β*	0.2
Migration time ago	*βN*
Migration probability	0.02
Mutation rate	2.5 × 10^−7^
Recombination rate	2.0 × 10^−7^
