# Supplementary material for: IntroUNET: identifying introgressed alleles via semantic segmentation
Source: bioRxiv. 2024 Jan 23:2023.02.07.527435. Originally published 2023 Feb 7. Preprint. [Version 3] doi: 10.1101/2023.02.07.527435 (PMC9979274; doi:10.1101/2023.02.07.527435)
Supplement: Supplement 1 [file NIHPP2023.02.07.527435v3-supplement-1.pdf]

bioRxiv preprint doi: https://doi.org/10.1101/2023.02.07.527435; this version posted January 23, 2024. The copyright holder for this preprint (which was not certified by peer review) is the author/funder, who has granted bioRxiv a license to display the preprint in perpetuity. It is made available under aCC-BY 4.0 International license.

9 Supporting Information Legends

Figure S1: A diagram of the chosen residual block structure shown with the dimensions for the first convolution for an initial size of two-populations by 64 individuals by 128 polymorphisms.

Figure S2: Loss function value trajectories calculated on training and validation data for versions of `IntroUNET` with different values of the label smoothing strength parameter $alpha$. All tests were calculated on simulated examples of the simple bidirectional scenario described in the Methods. Note that for $alpha = 0.1$ training loss is higher than validation loss. This is because label smoothing is only applied during training, and smoothing increases loss by adding noise to the target y values.

Figure S3: Five randomly chosen example segmentations on simulations from our simple bidirectional introgression scenario. Each example shows the input alignments for the two populations (labeled "pop 1" and "pop 2" respectively), the true introgressed alleles for these two populations (labeled "pop 1 (y)" and "pop 2 (y)" respectively), the introgressed alleles inferred by `IntroUNET` ("pop 1 (pred)" and "pop 2 (pred)"), and `IntroUNET`'s inferred introgression probabilities (labelled "prob", and scaled according to the color bar shown with the third example). Alignments and introgressed histories, true and predicted, are shown in the same format as in Figure 1.

Figure S4: Loss function value trajectories calculated on training and validation data for versions of `IntroUNET` with increasing sample sizes (32, 64, and 128 individuals per subpopulation), training set sizes (1000, 10000, and 100000 alignments), and window sizes (64, 128, and 256 polymorphisms). All tests were calculated on simulated examples of the simple bidirectional scenario described in the Methods.

Figure S5: `IntroUNET`'s accuracy when the split and migration times may be misspecified. `IntroUNET` was trained on 25 different combinations of the population split time and the upper bound of the range of possible introgression times (with the lower bound always set to zero). These simulations were performed in the same manner as described for the simple bidirectional model in the Methods, with the exception of these two parameters. Each heatmap in this grid shows the accuracy of one version of `IntroUNET` on each of the 25 test sets, and the parameter combination used to train that network is marked by a circle. For example, if the true split time is $2N$ generations ago and the true split time is 0.1 times the split time, one can observe the impact of misspecification on accuracy by comparing the top-left value in the top-left heatmap (i.e. no misspecification in this case) to the top-left value of all other heatmaps in the figure, which experience varying degrees of misspecification.

bioRxiv preprint doi: https://doi.org/10.1101/2023.02.07.527435; this version posted January 23, 2024. The copyright holder for this preprint (which was not certified by peer review) is the author/funder, who has granted bioRxiv a license to display the preprint in perpetuity. It is made available under aCC-BY 4.0 International license.

Figure S6: Performance of `IntroUNET` on the task of identifying introgression on a dataset where introgression is occuring in only one direction, but `IntroUNET` was trained to detect bidirectional introgression. The test data here were the same as those examined in Figure 4A, but the network used to perform inference was the same as that used for Figure 4C.

Figure S7: The impact of direct selection on `IntroUNET`'s performance. The left column shows confusion matrices obtained when using the same trained network used for the simple bidirectional introgression scenario whose parameters are laid out in Table 1, and applied to data simulated under the same model but with introgressed nucleotides experiencing negative selection. The column on the right shows results when testing the same `IntroUNET` model on data where introgressed segments are positively selected. The values of s represent the selection coefficient per introgressed nucleotide. Note that the shading represents the number of examples in each entry of the confusion matrix rather than the fraction of examples.

Figure S8: Five randomly chosen example segmentations on simulations from our simple bidirectional introgression scenario with relatively strong positive selection acting on each introgressed nucleotide ($s = 1 \times 10^{-5}$, or ~ 0.095 for a 10 kb introgressed segment). Each example shows the input alignments for the two populations (labeled "pop 1" and "pop 2" respectively), the true introgressed alleles for these two populations (labeled "pop 1 (y)" and "pop 2 (y)" respectively), the introgressed alleles inferred by `IntroUNET` ("pop 1 (pred)" and "pop 2 (pred)"), and `IntroUNET`'s inferred introgression probabilities (labelled "prob"). Alignments and introgressed histories, true and predicted, are shown in the same format as in Figure 1.

Figure S9: The impact of background selection (BGS) on `IntroUNET`'s performance. The confusion matrix shows `IntroUNET`'s classification performance when applying the same trained network used for the simple bidirectional introgression scenario whose parameters are laid out in Table 1 to data simulated under the the *D. melanogaster* BGS model specified in the Methods. Note that the shading represents the number of examples in each entry of the confusion matrix rather than the fraction of examples.

Figure S10: The impact of recombination rate on `IntroUNET`'s accuracy under the *D. melanogaster* background selection model. The left panel shows each simulation's accuracy, averaged across classified pixels from the central 100 kb of the simulated chromosome, as a function of the average recombination rate in that same window. The right panel shows each simulation's accuracy, averaged across classified pixels from the central 100 kb of the simulated chromosome, as a function of the recombination rate averaged across the entire simulated 1 Mb chromosome.

Figure S11: Performance of `IntroUNET` on the task of identifying bidirectional introgression using unphased genotype data. The test data here were the same as those examined in Figure 4C, but here rather than

bioRxiv preprint doi: https://doi.org/10.1101/2023.02.07.527435; this version posted January 23, 2024. The copyright holder for this preprint (which was not certified by peer review) is the author/funder, who has granted bioRxiv a license to display the preprint in perpetuity. It is made available under aCC-BY 4.0 International license.

predicted which haplotypes are introgressed, `IntroUNET`, when given a matrix of diploid genotypes, infers which diploid individuals have at least one introgressed allele at a given polymorphic site.

Figure S12: Diagram of the ghost introgression demographic model from [40], in which an unsampled archaic population splits off from the main population, before a pulse introgression event introduces alleles from this population into a sampled "Target" population, not an unsampled "Reference" population.

Figure S13: Ten example segmentations on simulations from our archaic introgression scenario: 5 from `IntroUNET` (left) and 5 from `ArchIE` (right). For each example we show the true and inferred introgressed alleles in the recipient population. For each method, both examples with and without introgression are shown.

Figure S14: Five example segmentations on simulations from our *Drosophila* introgression scenario. Each example shows the input alignments for the two populations, the true and inferred introgressed alleles for the *D. sechellia* population, and `IntroUNET`'s inferred introgression probabilities. Alignments and introgressed histories, true and predicted, are shown in the same format as in Figure 1.

Figure S15: Calibration curves showing the impact of Platt scaling on the accuracy of the introgression probability estimates produced by `IntroUNET`, calculated on the validation set from the *Drosophila* simulations (Methods). A) The fraction of alleles falling within a given bin of `IntroUNET`'s predicted probability of introgression that were in fact truly introgressed, prior to Platt recalibration. B) Same as (A), after recalibration.

Figure S16: Alignments and segmentations from six windows on chr3R in the vicinity of the locus of adaptive introgression (AI). Three of the windows (left) are from outside of the region affected by AI, and the other three (right) are within the AI locus. Each example shows the input alignments for the two populations (labeled "$\mathbf{X}$ (*dsim*)" and "$\mathbf{X}$ (*dsech*)", respectively), `IntroUNET`'s inferred introgression probabilities ("$\hat{y}$ (probs)"), and the most probable class for each allele in each individual ("$\hat{y}$ (class)"). Inferred introgressed histories and introgression probabilities are shown in the same format as in Figure 1. Alignments are shown in the same format as for previous figures, with the exception that for *D. sechellia* which has a different color scheme in haplotypes that were inferred to be introgressed in order to highlight these regions of the alignment: blue for the ancestral allele, and red for the derived allele.

Figure S17: The lengths of introgressed haplotypes predicted by `IntroUNET` at increasing distances away from the adaptive introgression even on chr3R. Introgressed haplotypes were defined as runs of consecutive SNPs classified as introgressed for a given individual. The sweep center was set to position 4624900, the center of the window with the lowest level of diversity in this region (data from [39]).

bioRxiv preprint doi: https://doi.org/10.1101/2023.02.07.527435; this version posted January 23, 2024. The copyright holder for this preprint (which was not certified by peer review) is the author/funder, who has granted bioRxiv a license to display the preprint in perpetuity. It is made available under aCC-BY 4.0 International license.

Figure S18: Results of training the same architecture on data seriated via different distance metrics, as well as using unsorted data (i.e. individuals within each population are arranged in the arbitrary order produced by the simulator), for the ghost-introgression problem (top row, panels A and B) and the *Drosophila* demographic model (bottom row, panels C and D). These plots show the values of training (A and C) and validation (B and D) loss over the course of training. Validation loss is usually lower than training in the case of *Drosophila* because label smoothing was applied to the training data for the purposes of regularization, but not to the validation data.

Figure S19: Confusion matrix showing performance of a classifier that detects genomic windows that have experienced introgression, trained and evaluated on data simulated under the *D. simulans*-*D. sechellia* scenario as described in the Methods.

Table S1: Bootstrap parameter estimates for the *D. simulans* and *D. sechellia* joint demographic model obtained via ∂a∂i. The parameters of the model are the ancestral population size (N_{ref}), the final population sizes of *D. sechellia* and *D. simulans* (N_{0-sech} and N_{0-sim}), the initial population sizes (N_{sech} and N_{sim}), the population split time (t_s), and the backwards migration rates ($m_{sim\rightarrow sech}$ and $m_{sech\rightarrow sim}$). Note that parameter estimates are shown for each bootstrap replicate for which our optimization procedure succeeded (Methods), but only those with log-likelihood scores greater than −1750 were used to simulate training data.

Table S2: CPU / GPU time estimates for accomplishing the experiments in the paper. The simulation and formatting results for this table were computed from a small sample of 430 replicates over 4 cores of an Intel Core i9-9900K CPU @ 3.60GHz and then scaled to give estimates for 10^5 replicates in each case. The Formatting column lists the time estimates for alignment sorting (for `IntroUNET`) and statistic calculation (for `ArchIE`). The Discriminator and Segmenter columns list the training times for classifying entire windows and for identifying introgressed haplotypes, respectively. In the GPU columns the estimate is simply the run time for the training described. The training of the neural networks was done on an NVIDIA A40 GPU, and we found that VRAM usage was <12Gb in all cases. We note that the `ArchIE` method computes statistics over the entire simulated window (224.64 on average in our simulated 50kb windows) whereas our method only formats a small sequential sample of SNPs from each replicate (192 for the Archaic introgression program). Below the time estimates for simulation are the simulated region size, and below the formatting times are the resulting "image" size or (populations, individuals, sites) and for the case of `ArchIE`, the window size in base pairs. Note that we do not include the time for execution on data after training, but we observed that classification times for all are generally negligible (although the sorting/statistic calculation steps must be performed first and these can be costly as shown in the Formatting section).

bioRxiv preprint doi: https://doi.org/10.1101/2023.02.07.527435; this version posted January 23, 2024. The copyright holder for this preprint (which was not certified by peer review) is the author/funder, who has granted bioRxiv a license to display the preprint in perpetuity. It is made available under aCC-BY 4.0 International license.

1257 # 10 Supporting Information

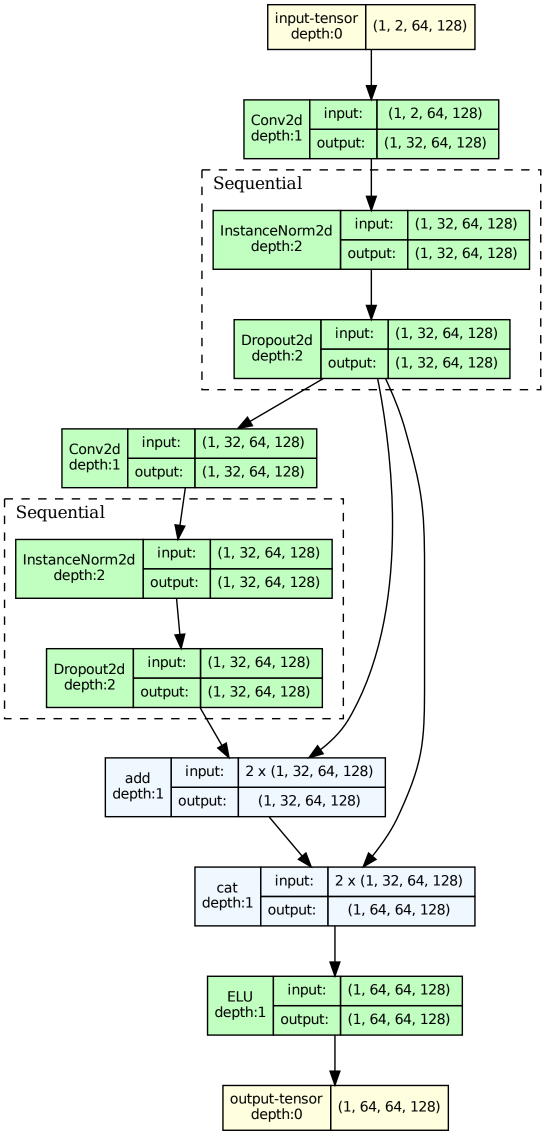

Figure S1: A diagram of the chosen residual block structure shown with the dimensions for the first convolution for an initial size of two-populations by 64 individuals by 128 polymorphisms.

bioRxiv preprint doi: https://doi.org/10.1101/2023.02.07.527435; this version posted January 23, 2024. The copyright holder for this preprint (which was not certified by peer review) is the author/funder, who has granted bioRxiv a license to display the preprint in perpetuity. It is made available under aCC-BY 4.0 International license.

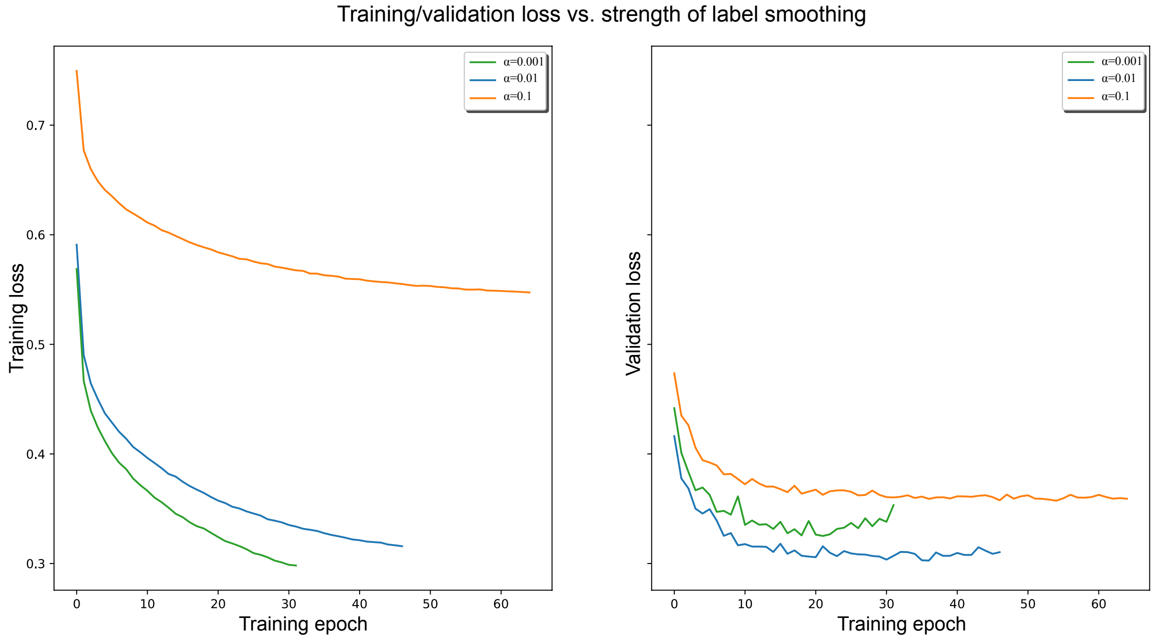

Figure S2: Loss function value trajectories calculated on training and validation data for versions of `IntroUNET` with different values of the label smoothing strength parameter $alpha$. All tests were calculated on simulated examples of the simple bidirectional scenario described in the Methods. Note that for $alpha = 0.1$ training loss is higher than validation loss. This is because label smoothing is only applied during training, and smoothing increases loss by adding noise to the target y values.

bioRxiv preprint doi: https://doi.org/10.1101/2023.02.07.527435; this version posted January 23, 2024. The copyright holder for this preprint (which was not certified by peer review) is the author/funder, who has granted bioRxiv a license to display the preprint in perpetuity. It is made available under aCC-BY 4.0 International license.

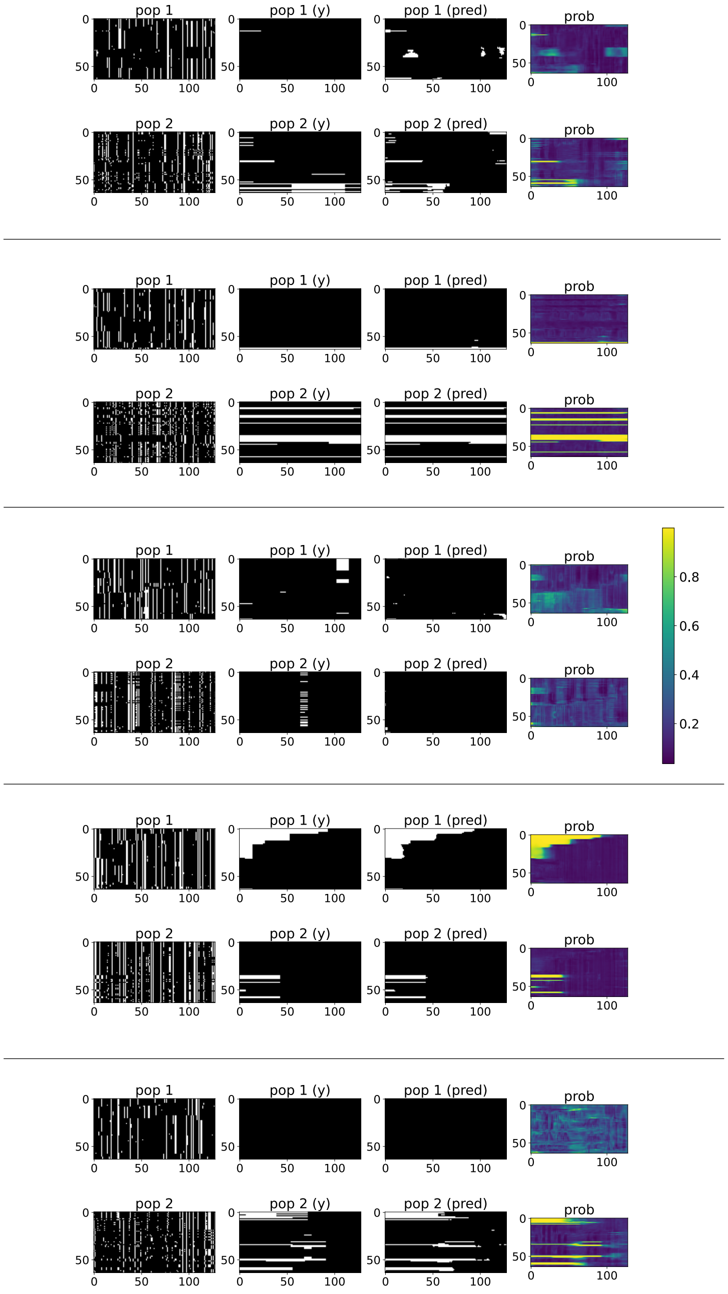

Figure S3: Five randomly chosen example segmentations on simulations from our simple bidirectional introgression scenario. Each example shows the input alignments for the two populations (labeled “pop 1” and “pop 2” respectively), the true introgressed alleles for these two populations (labeled “pop 1 (y)” and “pop 2 (y)” respectively), the introgressed alleles inferred by `IntroUNET` (“pop 1 (pred)” and “pop 2 (pred)”), and `IntroUNET`’s inferred introgression probabilities (labelled “prob”, and scaled according to the color bar shown with the third example). Alignments and introgressed histories, true and predicted, are shown in the same format as in Figure 1.

bioRxiv preprint doi: https://doi.org/10.1101/2023.02.07.527435; this version posted January 23, 2024. The copyright holder for this preprint (which was not certified by peer review) is the author/funder, who has granted bioRxiv a license to display the preprint in perpetuity. It is made available under aCC-BY 4.0 International license.

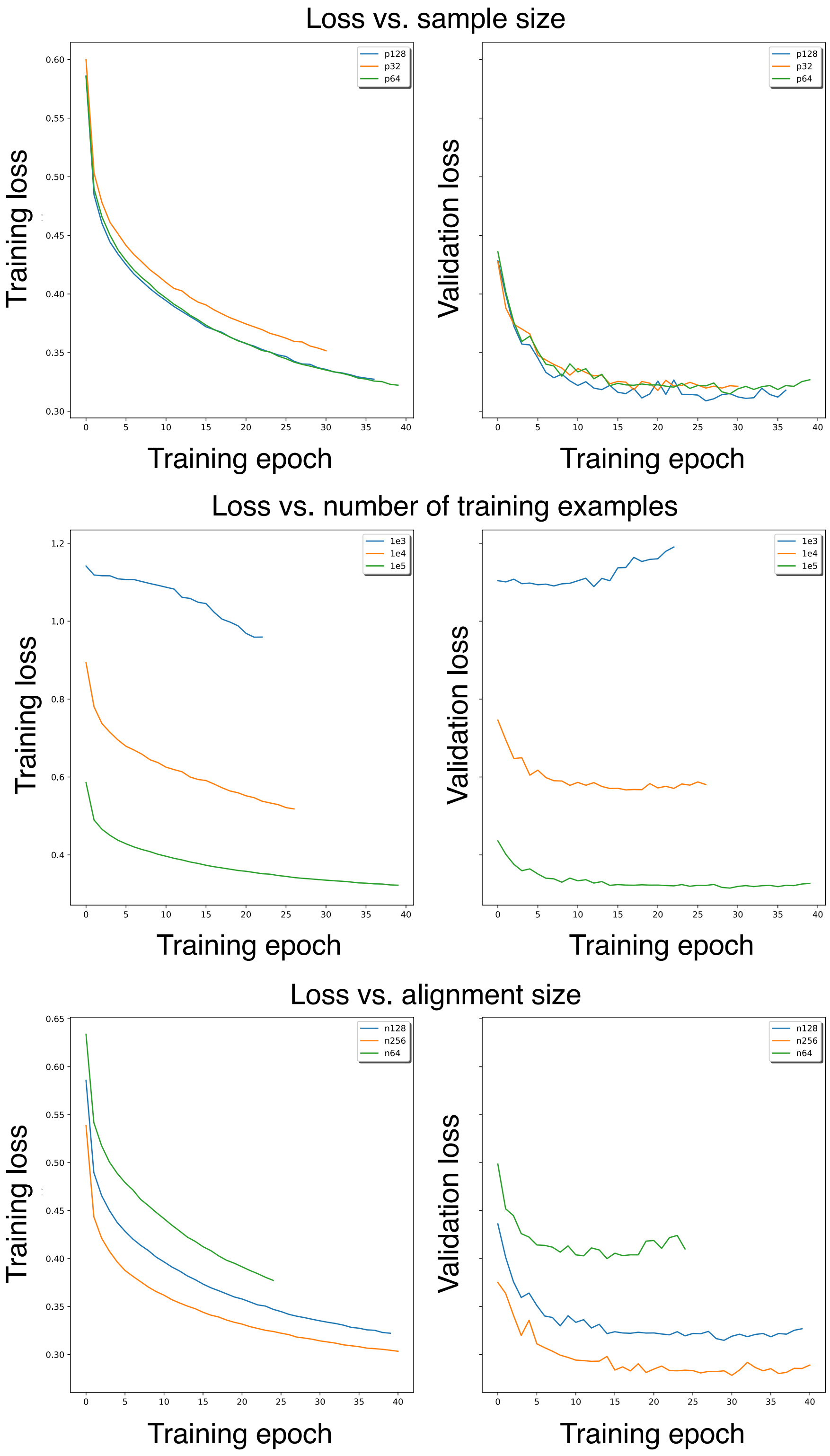

Figure S4: Loss function value trajectories calculated on training and validation data for versions of `IntroUNET` with increasing sample sizes (32, 64, and 128 individuals per subpopulation), training set sizes (1000, 10000, and 100000 alignments), and window sizes (64, 128, and 256 polymorphisms). All tests were calculated on simulated examples of the simple bidirectional scenario described in the Methods.

bioRxiv preprint doi: https://doi.org/10.1101/2023.02.07.527435; this version posted January 23, 2024. The copyright holder for this preprint (which was not certified by peer review) is the author/funder, who has granted bioRxiv a license to display the preprint in perpetuity. It is made available under aCC-BY 4.0 International license.

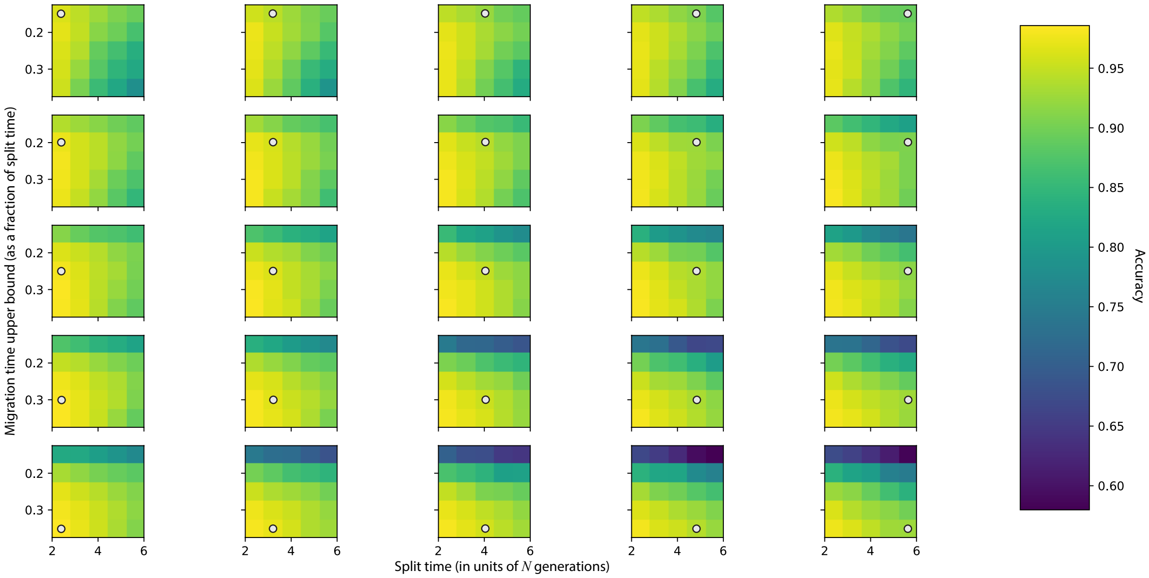

Figure S5: `IntroUNET`'s accuracy when the split and migration times may be misspecified. `IntroUNET` was trained on 25 different combinations of the population split time and the upper bound of the range of possible introgression times (with the lower bound always set to zero). These simulations were performed in the same manner as described for the simple bidirectional model in the Methods, with the exception of these two parameters. Each heatmap in this grid shows the accuracy of one version of `IntroUNET` on each of the 25 test sets, and the parameter combination used to train that network is marked by a circle. For example, if the true split time is $2N$ generations ago and the true split time is 0.1 times the split time, one can observe the impact of misspecification on accuracy by comparing the top-left value in the top-left heatmap (i.e. no misspecification in this case) to the top-left value of all other heatmaps in the figure, which experience varying degrees of misspecification.

bioRxiv preprint doi: https://doi.org/10.1101/2023.02.07.527435; this version posted January 23, 2024. The copyright holder for this preprint (which was not certified by peer review) is the author/funder, who has granted bioRxiv a license to display the preprint in perpetuity. It is made available under aCC-BY 4.0 International license.

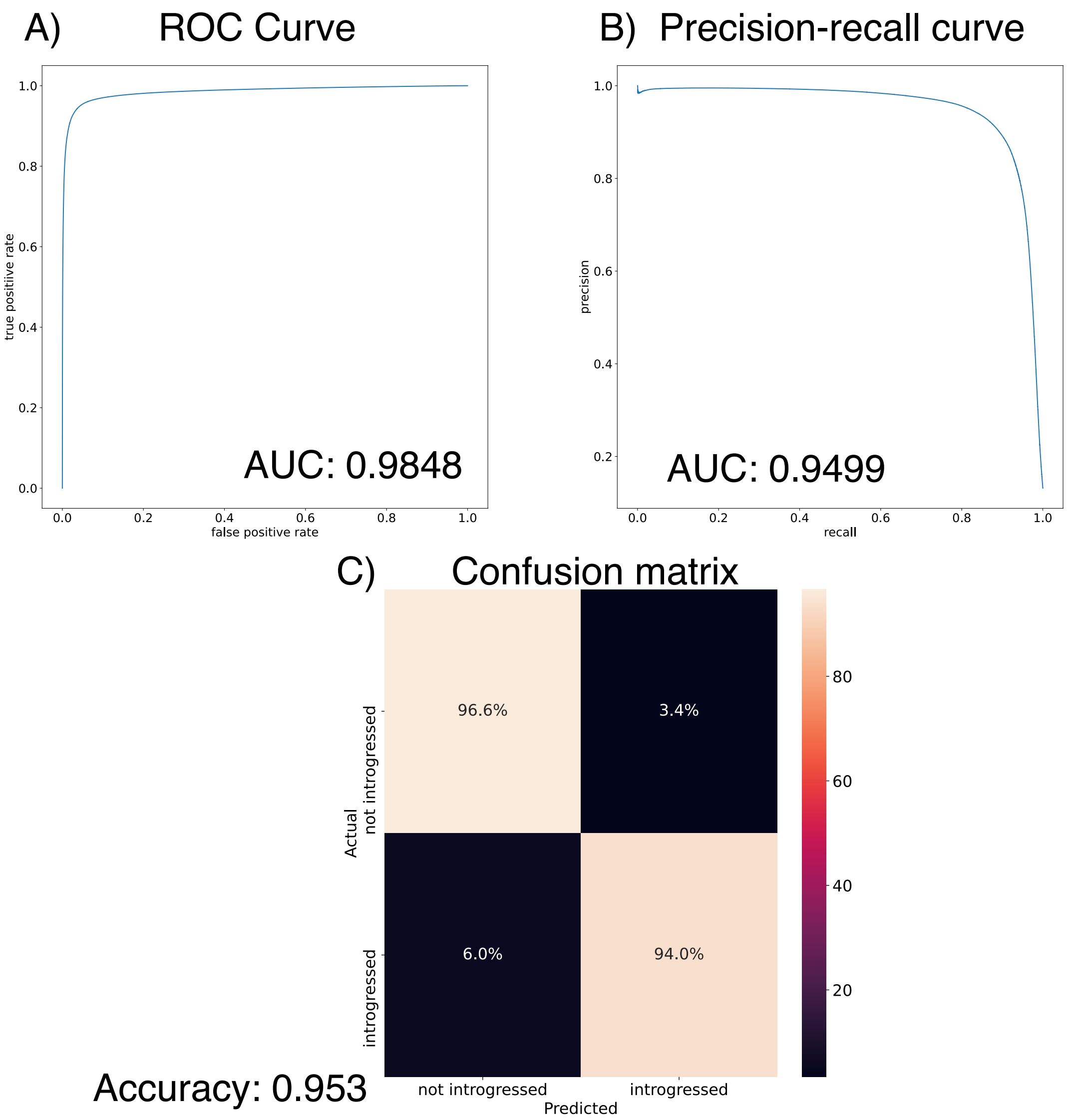

Figure S6: Performance of `IntroUNET` on the task of identifying introgression on a dataset where introgression is occuring in only one direction, but `IntroUNET` was trained to detect bidirectional introgression. The test data here were the same as those examined in Figure 4A, but the network used to perform inference was the same as that used for Figure 4C.

bioRxiv preprint doi: https://doi.org/10.1101/2023.02.07.527435; this version posted January 23, 2024. The copyright holder for this preprint (which was not certified by peer review) is the author/funder, who has granted bioRxiv a license to display the preprint in perpetuity. It is made available under aCC-BY 4.0 International license.

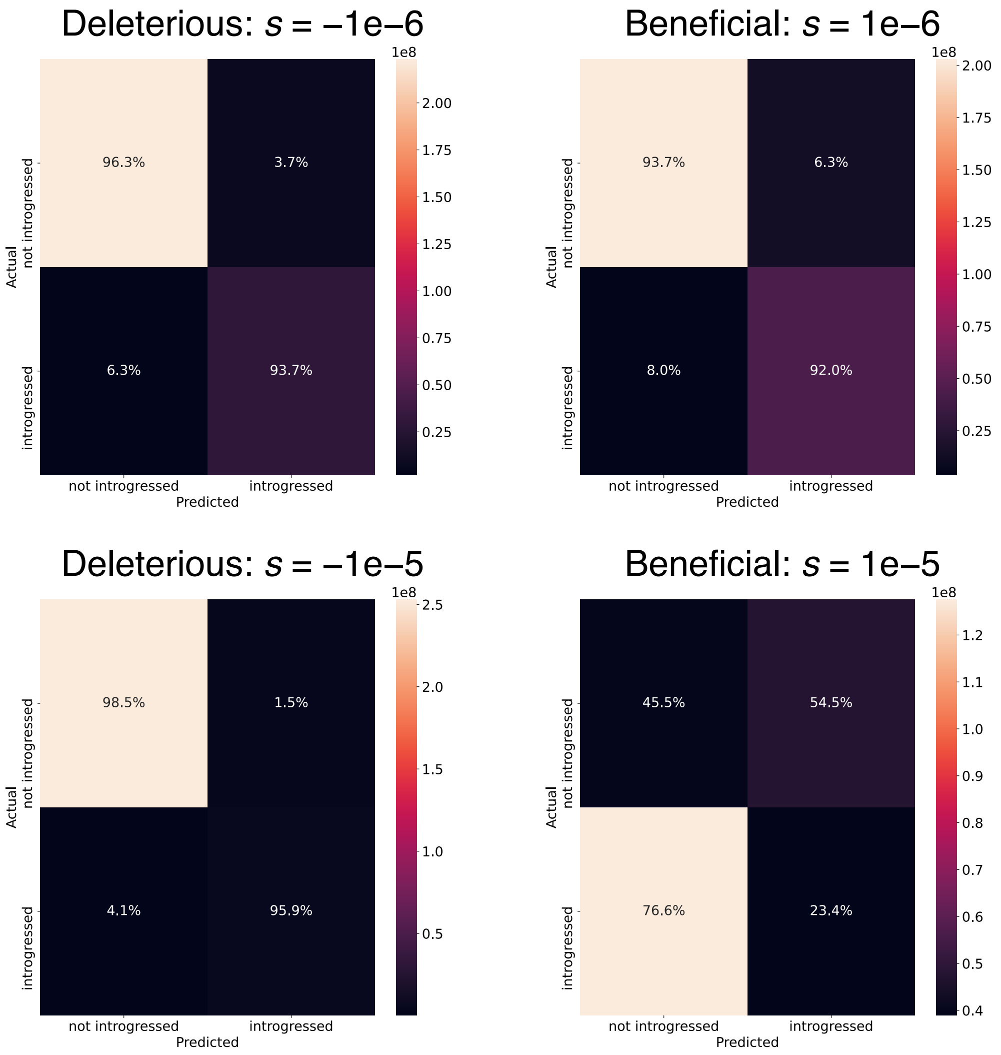

Figure S7: The impact of direct selection on `IntroUNET`'s performance. The left column shows confusion matrices obtained when using the same trained network used for the simple bidirectional introgression scenario whose parameters are laid out in Table 1, and applied to data simulated under the same model but with introgressed nucleotides experiencing negative selection. The column on the right shows results when testing the same `IntroUNET` model on data where introgressed segments are positively selected. The values of s represent the selection coefficient per introgressed nucleotide. Note that the shading represents the number of examples in each entry of the confusion matrix rather than the fraction of examples.

bioRxiv preprint doi: https://doi.org/10.1101/2023.02.07.527435; this version posted January 23, 2024. The copyright holder for this preprint (which was not certified by peer review) is the author/funder, who has granted bioRxiv a license to display the preprint in perpetuity. It is made available under aCC-BY 4.0 International license.

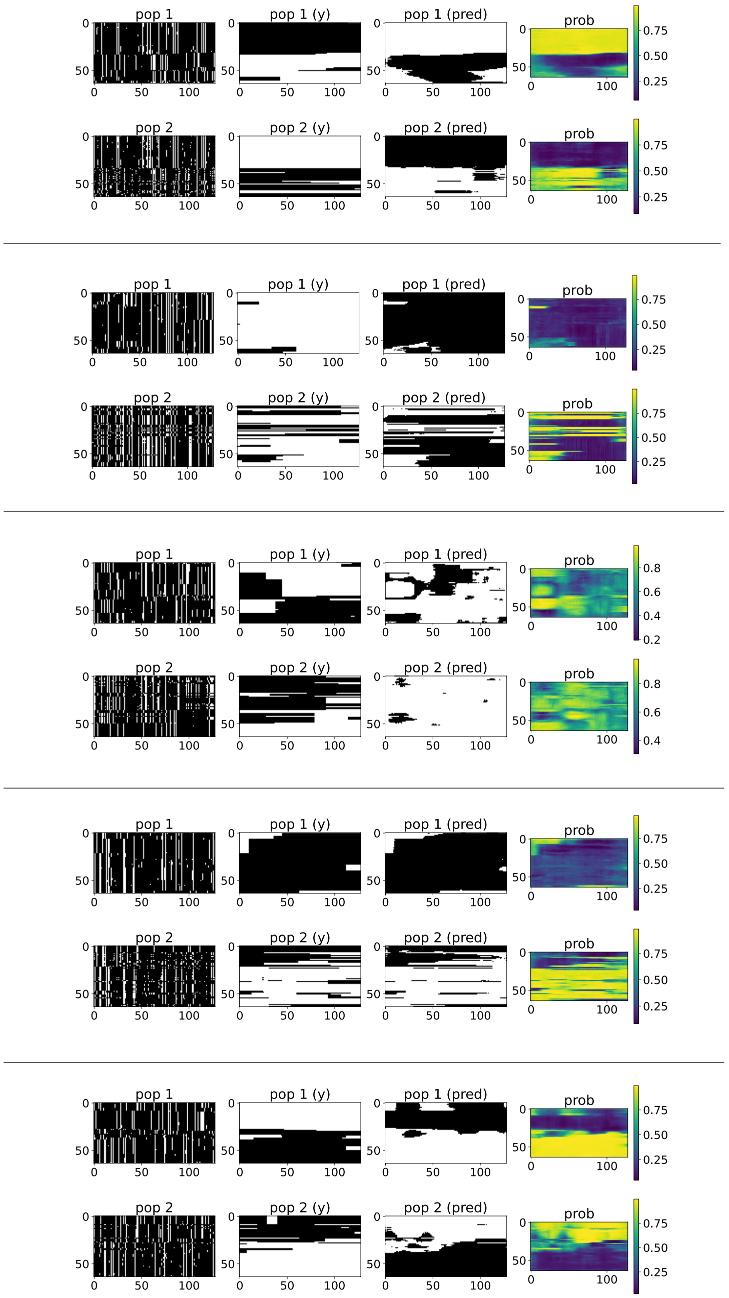

Figure S8: Five randomly chosen example segmentations on simulations from our simple bidirectional introgression scenario with relatively strong positive selection acting on each introgressed nucleotide ($s = 1 \times 10^{-5}$, or ~ 0.095 for a 10 kb introgressed segment). Each example shows the input alignments for the two populations (labeled "pop 1" and "pop 2" respectively), the true introgressed alleles for these two populations (labeled "pop 1 (y)" and "pop 2 (y)" respectively), the introgressed alleles inferred by `IntroUNET` ("pop 1 (pred)" and "pop 2 (pred)"), and `IntroUNET`'s inferred introgression probabilities (labelled "prob"). Alignments and introgressed histories, true and predicted, are shown in the same format as in Figure 1.

bioRxiv preprint doi: https://doi.org/10.1101/2023.02.07.527435; this version posted January 23, 2024. The copyright holder for this preprint (which was not certified by peer review) is the author/funder, who has granted bioRxiv a license to display the preprint in perpetuity. It is made available under aCC-BY 4.0 International license.

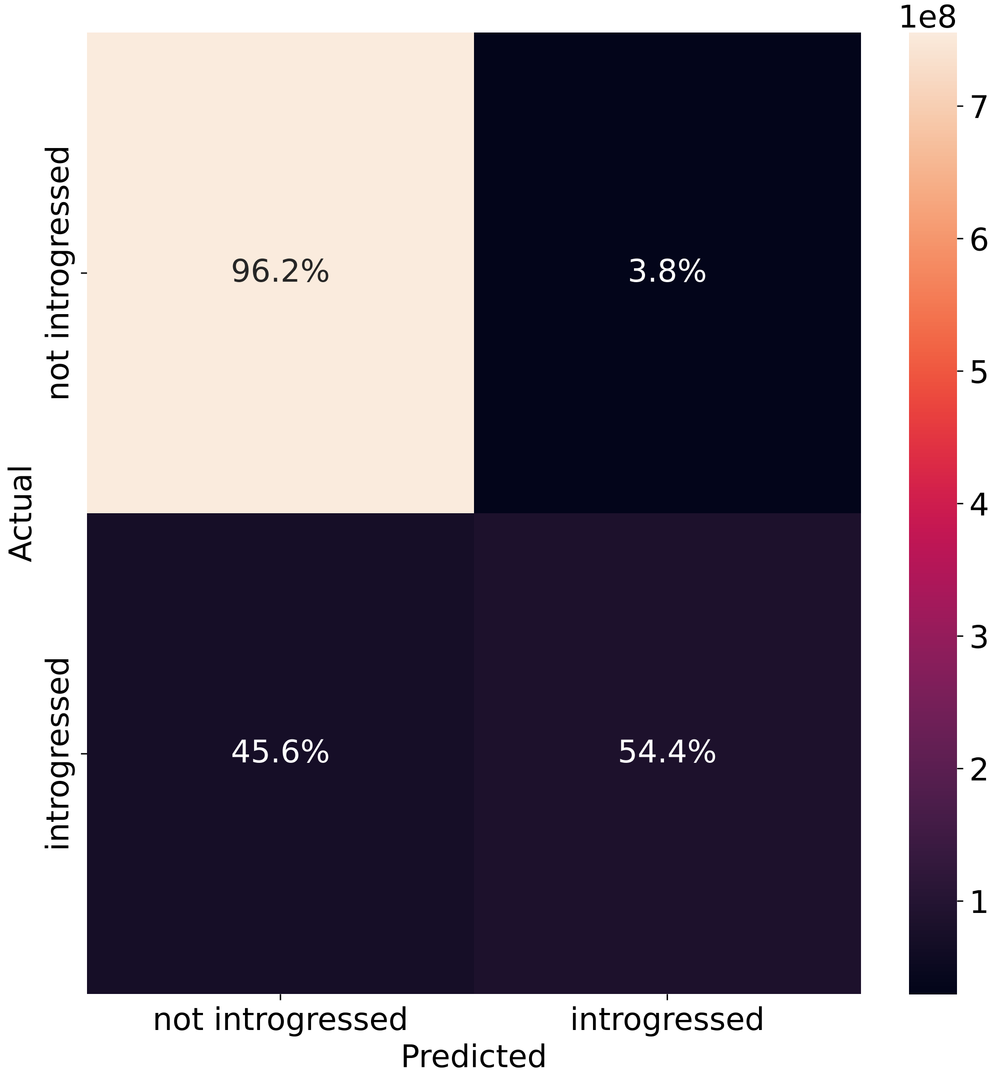

Figure S9: The impact of background selection (BGS) on `IntroUNET`'s performance. The confusion matrix shows `IntroUNET`'s classification performance when applying the same trained network used for the simple bidirectional introgression scenario whose parameters are laid out in Table 1 to data simulated under the the *D. melanogaster* BGS model specified in the Methods. Note that the shading represents the number of examples in each entry of the confusion matrix rather than the fraction of examples.

bioRxiv preprint doi: https://doi.org/10.1101/2023.02.07.527435; this version posted January 23, 2024. The copyright holder for this preprint (which was not certified by peer review) is the author/funder, who has granted bioRxiv a license to display the preprint in perpetuity. It is made available under aCC-BY 4.0 International license.

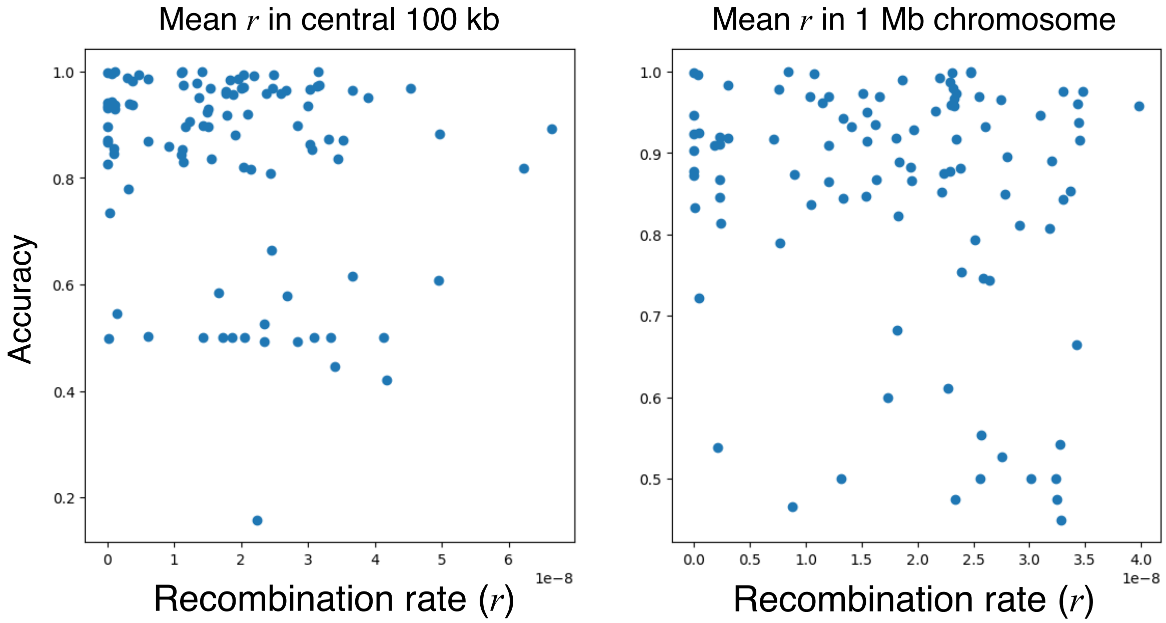

Figure S10: The impact of recombination rate on `IntroUNET`'s accuracy under the *D. melanogaster* background selection model. The left panel shows each simulation's accuracy, averaged across classified pixels from the central 100 kb of the simulated chromosome, as a function of the average recombination rate in that same window. The right panel shows each simulation's accuracy, averaged across classified pixels from the central 100 kb of the simulated chromosome, as a function of the recombination rate averaged across the entire simulated 1 Mb chromosome.

bioRxiv preprint doi: https://doi.org/10.1101/2023.02.07.527435; this version posted January 23, 2024. The copyright holder for this preprint (which was not certified by peer review) is the author/funder, who has granted bioRxiv a license to display the preprint in perpetuity. It is made available under aCC-BY 4.0 International license.

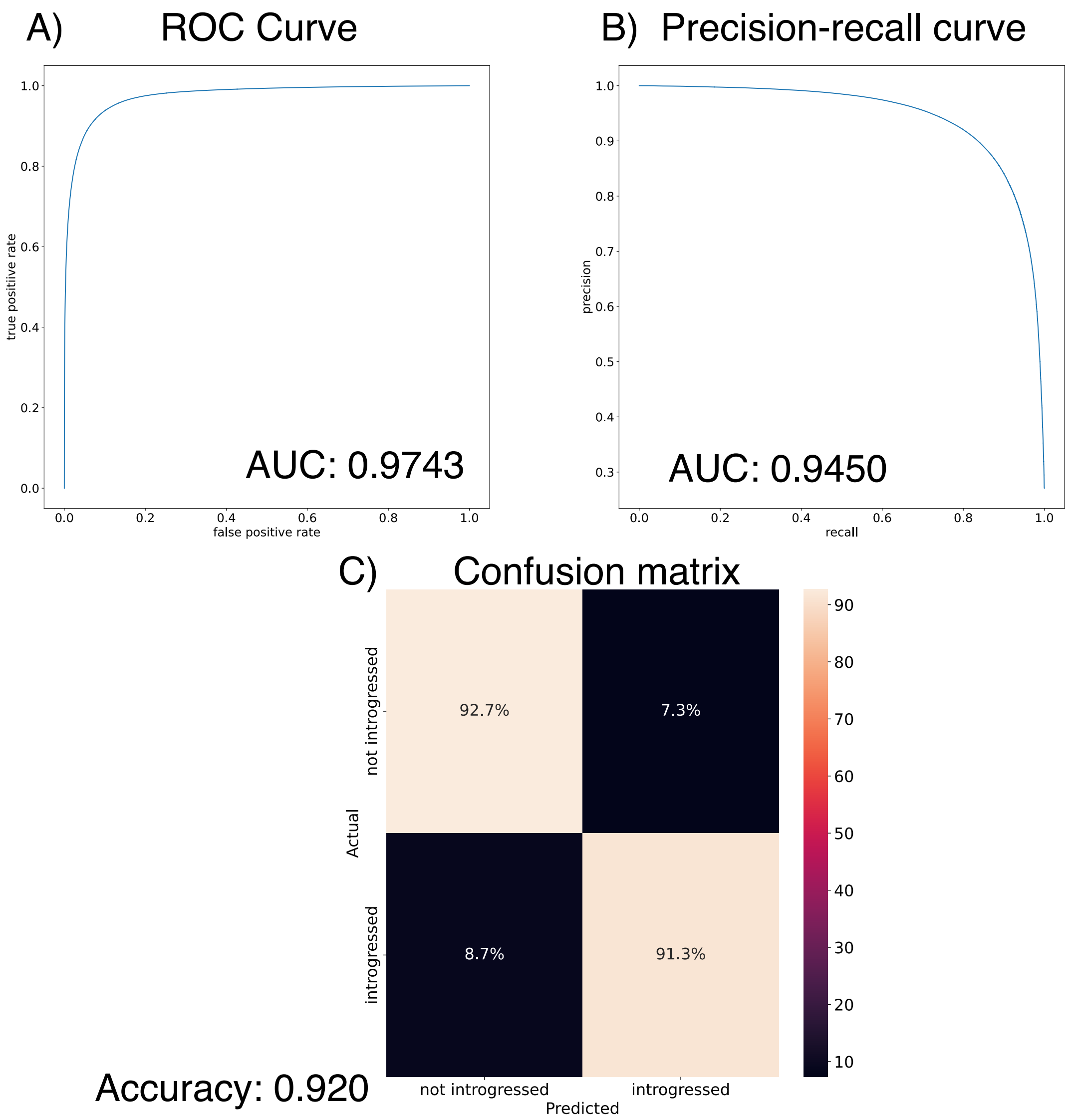

Figure S11: Performance of `IntroUNET` on the task of identifying bidirectional introgression using unphased genotype data. The test data here were the same as those examined in Figure 4C, but here rather than predicted which haplotypes are introgressed, `IntroUNET`, when given a matrix of diploid genotypes, infers which diploid individuals have at least one introgressed allele at a given polymorphic site.

bioRxiv preprint doi: https://doi.org/10.1101/2023.02.07.527435; this version posted January 23, 2024. The copyright holder for this preprint (which was not certified by peer review) is the author/funder, who has granted bioRxiv a license to display the preprint in perpetuity. It is made available under aCC-BY 4.0 International license.

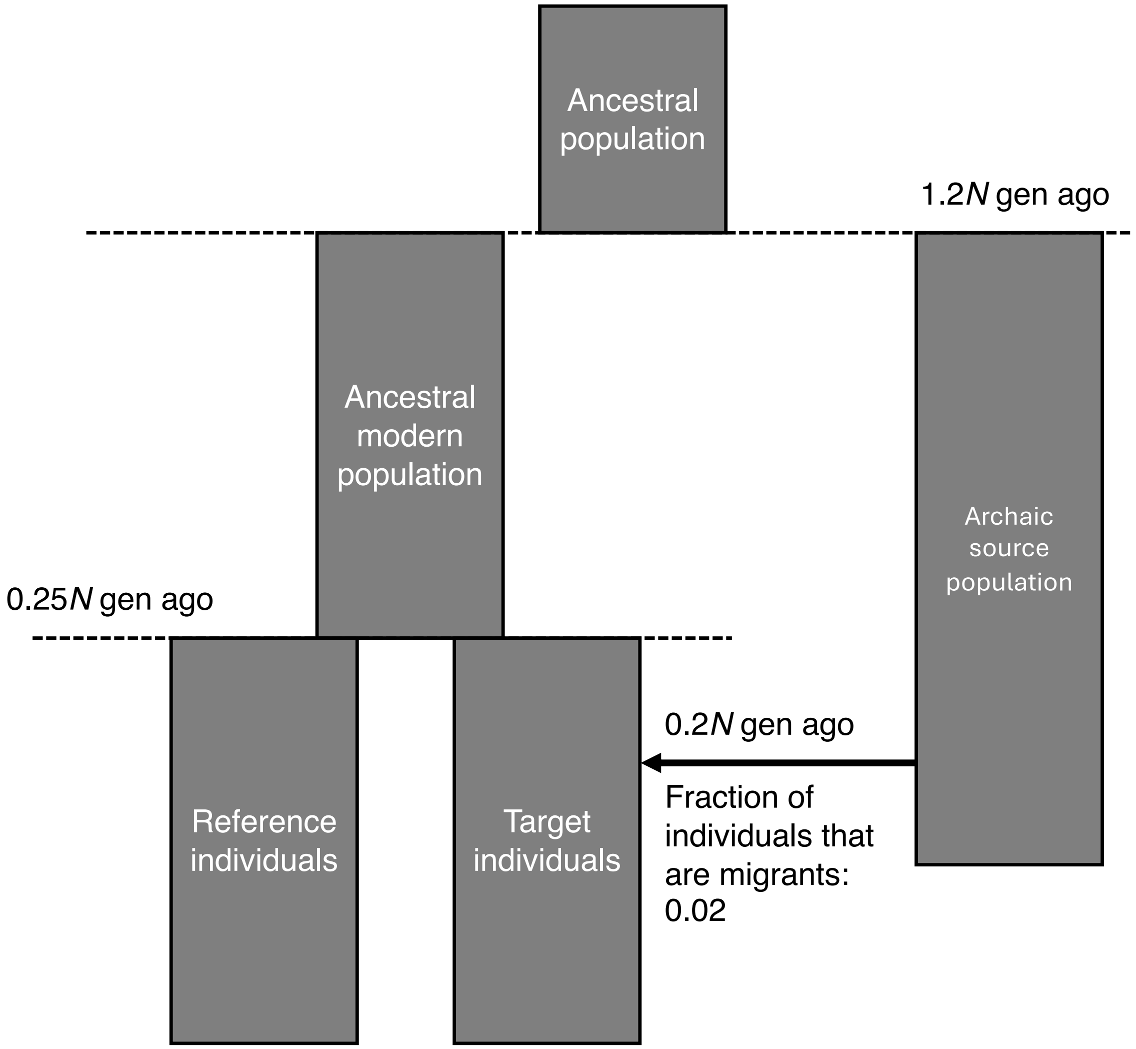

Figure S12: Diagram of the ghost introgression demographic model from [40], in which an unsampled archaic population splits off from the main population, before a pulse introgression event introduces alleles from this population into a sampled "Target" population, not an unsampled "Reference" population.

bioRxiv preprint doi: https://doi.org/10.1101/2023.02.07.527435; this version posted January 23, 2024. The copyright holder for this preprint (which was not certified by peer review) is the author/funder, who has granted bioRxiv a license to display the preprint in perpetuity. It is made available under aCC-BY 4.0 International license.

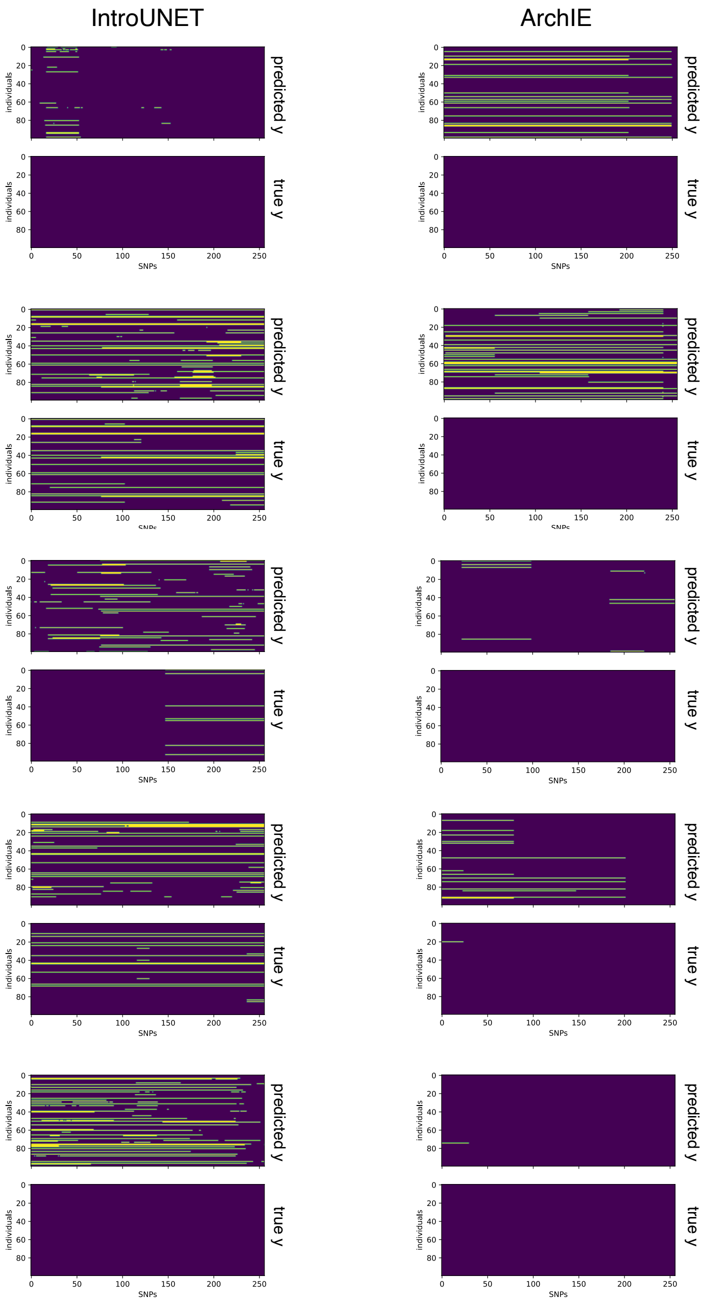

Figure S13: Ten example segmentations on simulations from our archaic introgression scenario: 5 from `IntroUNET` (left) and 5 from `ArchIE` (right). For each example we show the true and inferred introgressed alleles in the recipient population. For each method, both examples with and without introgression are shown.

bioRxiv preprint doi: https://doi.org/10.1101/2023.02.07.527435; this version posted January 23, 2024. The copyright holder for this preprint (which was not certified by peer review) is the author/funder, who has granted bioRxiv a license to display the preprint in perpetuity. It is made available under aCC-BY 4.0 International license.

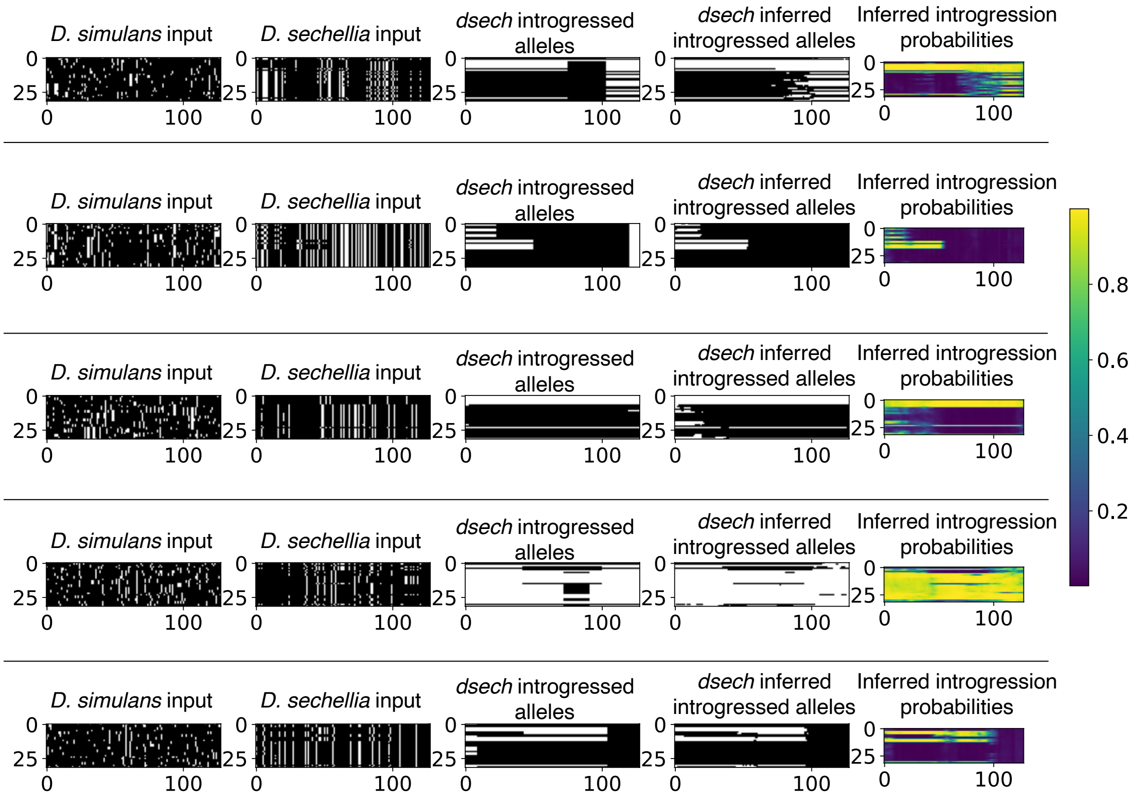

Figure S14: Five example segmentations on simulations from our *Drosophila* introgression scenario. Each example shows the input alignments for the two populations, the true and inferred introgressed alleles for the *D. sechellia* population, and `IntroUNET`'s inferred introgression probabilities. Alignments and introgressed histories, true and predicted, are shown in the same format as in Figure 1.

bioRxiv preprint doi: https://doi.org/10.1101/2023.02.07.527435; this version posted January 23, 2024. The copyright holder for this preprint (which was not certified by peer review) is the author/funder, who has granted bioRxiv a license to display the preprint in perpetuity. It is made available under aCC-BY 4.0 International license.

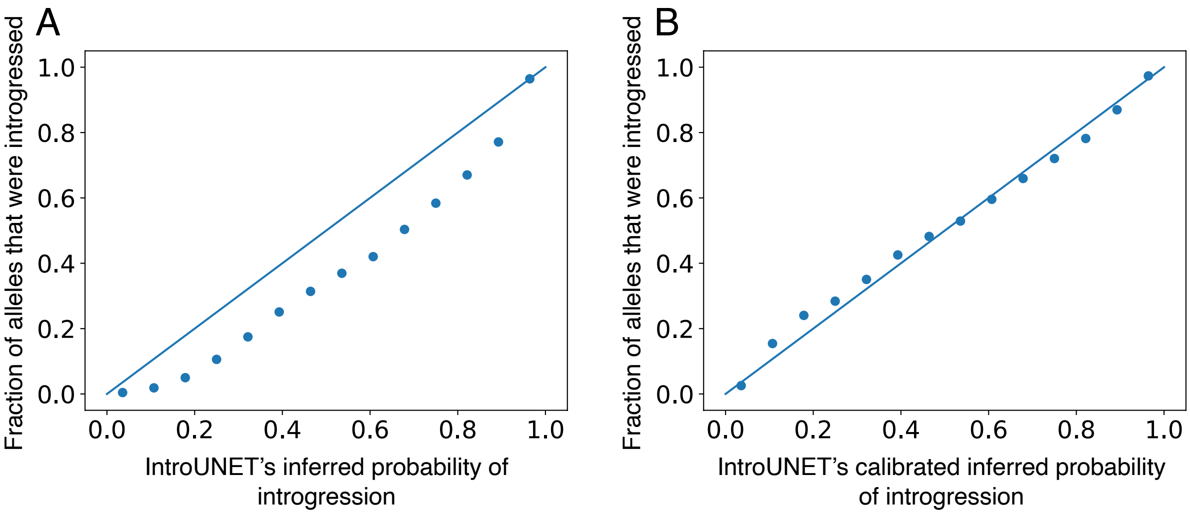

Figure S15: Calibration curves showing the impact of Platt scaling on the accuracy of the introgression probability estimates produced by `IntroUNET`, calculated on the validation set from the *Drosophila* simulations (Methods). A) The fraction of alleles falling within a given bin of `IntroUNET`'s predicted probability of introgression that were in fact truly introgressed, prior to Platt recalibration. B) Same as (A), after recalibration.

bioRxiv preprint doi: https://doi.org/10.1101/2023.02.07.527435; this version posted January 23, 2024. The copyright holder for this preprint (which was not certified by peer review) is the author/funder, who has granted bioRxiv a license to display the preprint in perpetuity. It is made available under aCC-BY 4.0 International license.

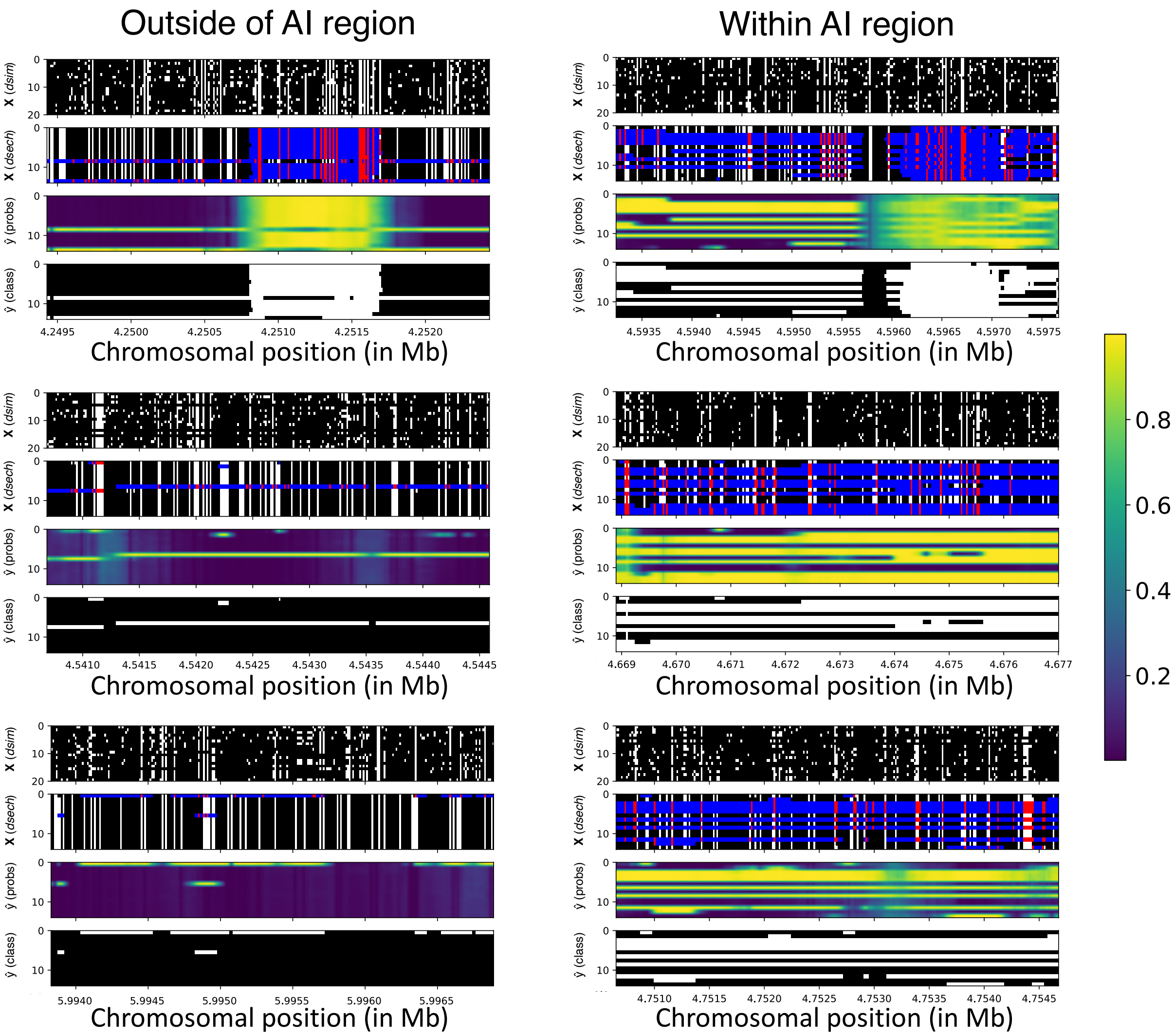

Figure S16: Alignments and segmentations from six windows on chr3R in the vicinity of the locus of adaptive introgression (AI). Three of the windows (left) are from outside of the region affected by AI, and the other three (right) are within the AI locus. Each example shows the input alignments for the two populations (labeled "**X** (*dsim*)" and "**X** (*dsech*)", respectively), `IntroUNET`'s inferred introgression probabilities ("$\hat{y}$ (probs)"), and the most probable class for each allele in each individual ("$\hat{y}$ (class)"). Inferred introgressed histories and introgression probabilities are shown in the same format as in Figure 1. Alignments are shown in the same format as for previous figures, with the exception that for *D. sechellia* which has a different color scheme in haplotypes that were inferred to be introgressed in order to highlight these regions of the alignment: blue for the ancestral allele, and red for the derived allele.

bioRxiv preprint doi: https://doi.org/10.1101/2023.02.07.527435; this version posted January 23, 2024. The copyright holder for this preprint (which was not certified by peer review) is the author/funder, who has granted bioRxiv a license to display the preprint in perpetuity. It is made available under aCC-BY 4.0 International license.

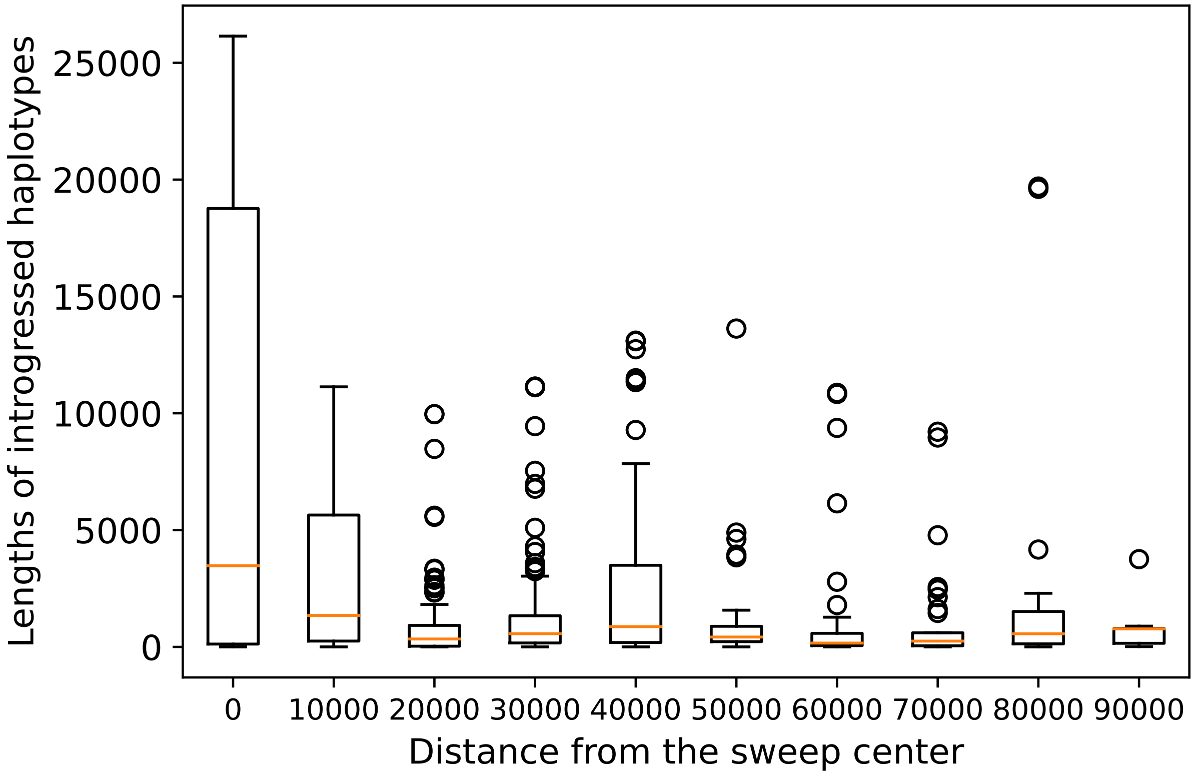

Figure S17: The lengths of introgressed haplotypes predicted by `IntroUNET` at increasing distances away from the adaptive introgression even on chr3R. Introgressed haplotypes were defined as runs of consecutive SNPs classified as introgressed for a given individual. The sweep center was set to position 4624900, the center of the window with the lowest level of diversity in this region (data from [39]).

bioRxiv preprint doi: https://doi.org/10.1101/2023.02.07.527435; this version posted January 23, 2024. The copyright holder for this preprint (which was not certified by peer review) is the author/funder, who has granted bioRxiv a license to display the preprint in perpetuity. It is made available under aCC-BY 4.0 International license.

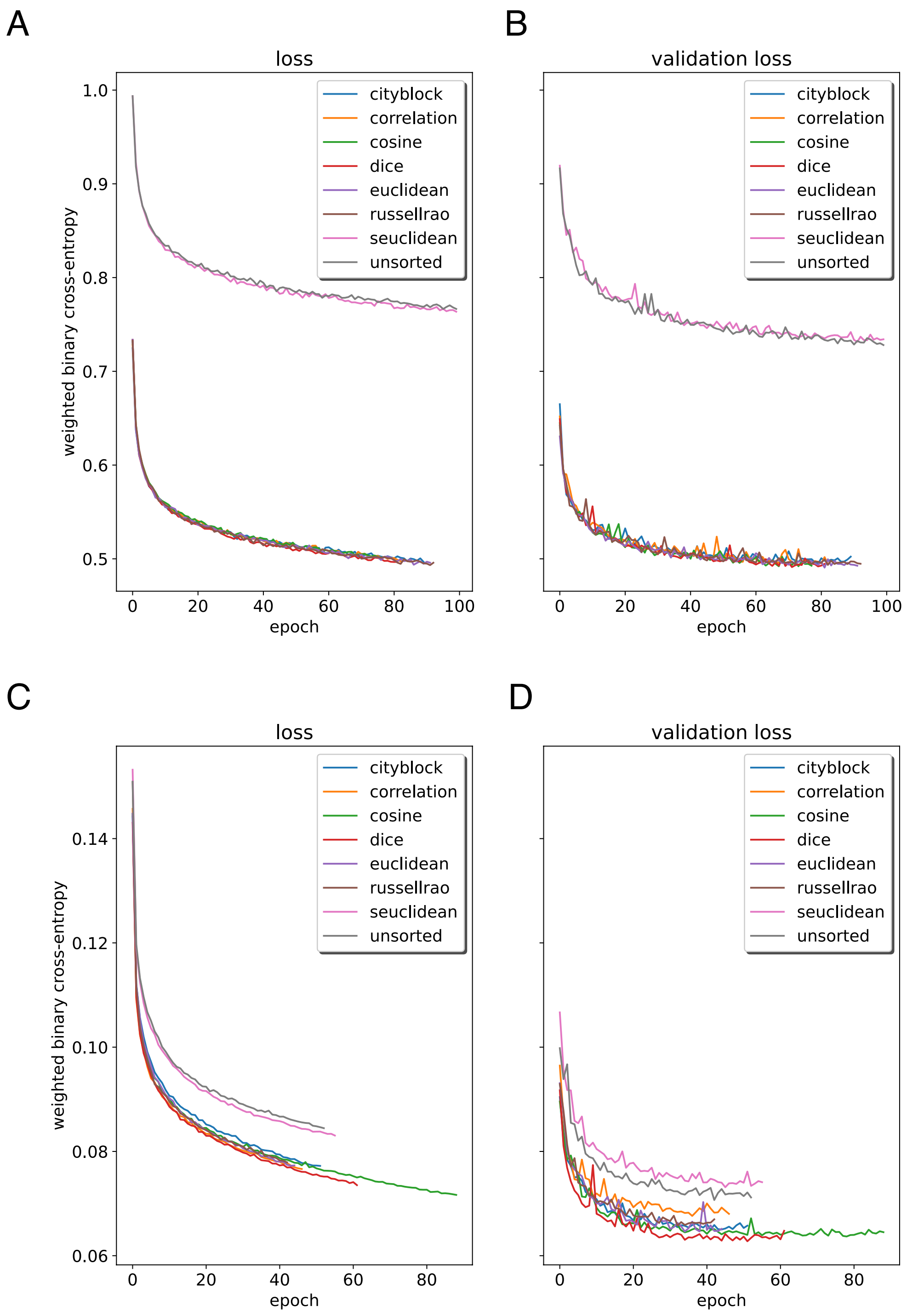

Figure S18: Results of training the same architecture on data seriated via different distance metrics, as well as using unsorted data (i.e. individuals within each population are arranged in the arbitrary order produced by the simulator), for the ghost-introgression problem (top row, panels A and B) and the *Drosophila* demographic model (bottom row, panels C and D). These plots show the values of training (A and C) and validation (B and D) loss over the course of training. Validation loss is usually lower than training in the case of *Drosophila* because label smoothing was applied to the training data for the purposes of regularization, but not to the validation data.

bioRxiv preprint doi: https://doi.org/10.1101/2023.02.07.527435; this version posted January 23, 2024. The copyright holder for this preprint (which was not certified by peer review) is the author/funder, who has granted bioRxiv a license to display the preprint in perpetuity. It is made available under aCC-BY 4.0 International license.

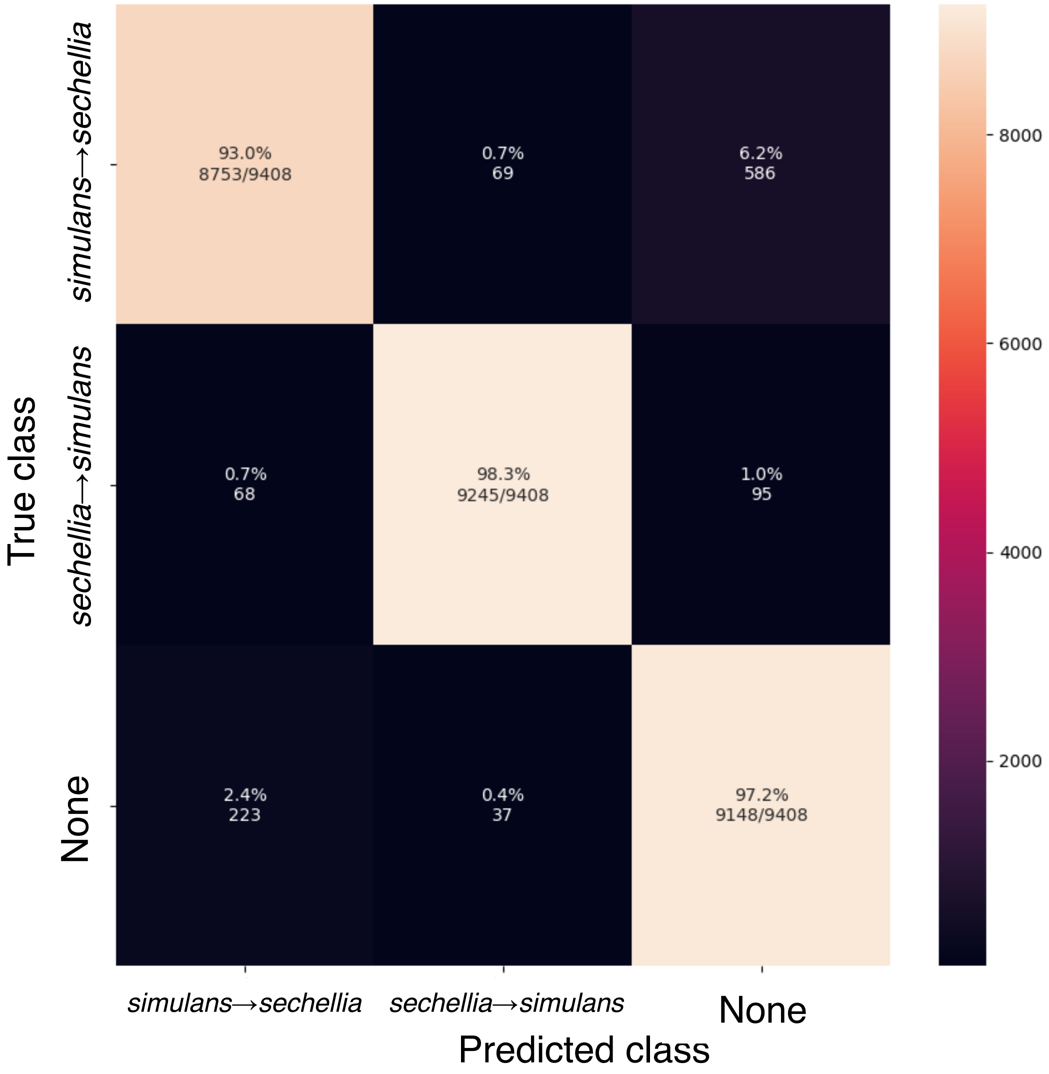

Figure S19: Confusion matrix showing performance of a classifier that detects genomic windows that have experienced introgression, trained and evaluated on data simulated under the *D. simulans*-*D. sechellia* scenario as described in the Methods.

bioRxiv preprint doi: https://doi.org/10.1101/2023.02.07.527435; this version posted January 23, 2024. The copyright holder for this preprint (which was not certified by peer review) is the author/funder, who has granted bioRxiv a license to display the preprint in perpetuity. It is made available under aCC-BY 4.0 International license.

Log-likelihood	AIC	N_{ref}	N_{0-sech}	N_{0-sim}	N_{sech}	N_{sim}	t_s	$m_{sim\rightarrow sech}$	$m_{sech\rightarrow sim}$
-1.54e+03	3.1e+03	3.27e+05	7.47e+04	3.27e+06	5.94e+06	1.55e+04	5.31e+04	0.0104	0.141
-1.42e+03	2.85e+03	2.86e+05	7.29e+04	2.86e+06	5.55e+06	1.72e+04	5.11e+04	0.0136	0.061
-1.27e+03	2.56e+03	2.94e+05	7.82e+04	2.94e+06	5.79e+06	1.75e+04	5.4e+04	0.0138	0.05
-1.28e+03	2.58e+03	3.04e+05	7.81e+04	3.04e+06	5.46e+06	1.79e+04	5.36e+04	0.0143	0.0648
-1.26e+03	2.54e+03	2.88e+05	7.42e+04	2.88e+06	5.14e+06	1.73e+04	5.12e+04	0.0133	0.0595
-1.27e+03	2.55e+03	3.12e+05	8.1e+04	3.12e+06	5.7e+06	1.8e+04	5.53e+04	0.0141	0.0694
-2.99e+03	5.99e+03	3.56e+04	2.54e+04	2.02e+05	3.43e+06	1.84e+04	9.49e+04	0.00216	0.0204
-1.15e+03	2.32e+03	2.91e+05	7.67e+04	2.91e+06	5.16e+06	1.65e+04	5.24e+04	0.0129	0.0528
-1.07e+03	2.15e+03	2.84e+05	7.16e+04	2.84e+06	5.16e+06	1.62e+04	5.06e+04	0.0132	0.0589
-1.64e+03	3.29e+03	2.91e+05	7.25e+04	2.93e+05	5.15e+06	2.06e+04	5.51e+04	0.0116	0.103
-1.4e+03	2.8e+03	3.04e+05	7.6e+04	3.04e+06	5.32e+06	1.72e+04	5.31e+04	0.0136	0.0734
-2.98e+03	5.97e+03	3.64e+04	2.06e+04	2.25e+05	3.48e+06	1.77e+04	9.71e+04	0.00216	0.0239
-1.2e+03	2.41e+03	2.91e+05	7.23e+04	2.9e+06	5.05e+06	1.66e+04	5.09e+04	0.0132	0.078
-1.11e+03	2.23e+03	3.04e+05	8.15e+04	3.04e+06	5.67e+06	1.73e+04	5.62e+04	0.0137	0.0675
-1.25e+03	2.51e+03	2.94e+05	7.71e+04	2.94e+06	5.5e+06	1.72e+04	5.38e+04	0.013	0.0571
-1.31e+03	2.64e+03	2.95e+05	7.98e+04	2.95e+06	5.42e+06	1.69e+04	5.34e+04	0.0134	0.0529
-1.14e+03	2.29e+03	2.73e+05	6.64e+04	2.73e+06	5.14e+06	1.56e+04	4.78e+04	0.0131	0.0573
-1.23e+03	2.48e+03	2.95e+05	7.16e+04	2.95e+06	5.44e+06	1.72e+04	5.06e+04	0.0137	0.0626
-3.13e+03	6.27e+03	3.62e+04	2.42e+04	2.84e+05	3.46e+06	1.79e+04	9.44e+04	0.00223	0.0225
-2.96e+03	5.94e+03	4.24e+04	2.53e+04	2.99e+05	3.58e+06	1.84e+04	1.01e+05	0.00247	0.0255
-2.89e+03	5.79e+03	3.95e+04	2.42e+04	6.15e+04	3.6e+06	1.79e+04	9.48e+04	0.0024	0.0213
-1.24e+03	2.49e+03	2.94e+05	7.79e+04	2.94e+06	5.82e+06	1.66e+04	5.38e+04	0.013	0.0662
-1.43e+03	2.88e+03	2.98e+05	7.5e+04	2.98e+06	5.47e+06	1.77e+04	5.17e+04	0.0144	0.0688
-1.29e+03	2.6e+03	3.08e+05	7.78e+04	3.08e+06	5.69e+06	1.74e+04	5.55e+04	0.0132	0.0848
-1.33e+03	2.66e+03	3.01e+05	7.75e+04	3.01e+06	5.66e+06	1.74e+04	5.3e+04	0.0137	0.0567
-1.27e+03	2.56e+03	2.87e+05	7.73e+04	2.87e+06	5.05e+06	1.66e+04	5.19e+04	0.0133	0.0649
-2.88e+03	5.78e+03	3.87e+04	2.57e+04	3.87e+05	3.44e+06	1.86e+04	9.52e+04	0.00236	0.0231
-1.31e+03	2.62e+03	3e+05	7.83e+04	3e+06	5.68e+06	1.71e+04	5.43e+04	0.0136	0.0767
-1.26e+03	2.53e+03	3.05e+05	8.19e+04	3.05e+06	5.85e+06	1.78e+04	5.47e+04	0.0135	0.0542
-1.34e+03	2.69e+03	3.01e+05	7.67e+04	3.01e+06	5.65e+06	1.75e+04	5.31e+04	0.0134	0.0666
-1.35e+03	2.72e+03	2.94e+05	7.76e+04	2.94e+06	5.47e+06	1.72e+04	5.29e+04	0.0131	0.0654
-2.68e+03	5.38e+03	3.72e+04	2.5e+04	3.72e+05	3.72e+06	1.91e+04	9.91e+04	0.00239	0.0189
-1.28e+03	2.57e+03	2.81e+05	7.61e+04	2.81e+06	5.43e+06	1.66e+04	5.15e+04	0.0134	0.0642
-2.84e+03	5.69e+03	3.66e+04	2.58e+04	3.11e+05	3.52e+06	1.79e+04	9.72e+04	0.00221	0.0195
-1.18e+03	2.36e+03	2.93e+05	7.66e+04	2.93e+06	5.32e+06	1.65e+04	5.23e+04	0.0134	0.0628
-1.26e+03	2.54e+03	2.9e+05	7.13e+04	2.9e+06	5.42e+06	1.65e+04	5.01e+04	0.0133	0.0538
-3e+03	6.01e+03	4.94e+04	2.75e+04	4.94e+05	3.29e+06	1.74e+04	9.03e+04	0.00318	0.028
-1.24e+03	2.49e+03	2.92e+05	7.67e+04	2.92e+06	5.66e+06	1.74e+04	5.24e+04	0.0139	0.0415
-1.35e+03	2.71e+03	3.04e+05	7.74e+04	3.04e+06	5.33e+06	1.7e+04	5.32e+04	0.0138	0.0694
-2.51e+03	5.04e+03	3.57e+04	2.37e+04	1.51e+05	3.47e+06	1.66e+04	9.52e+04	0.00231	0.0225
-1.23e+03	2.48e+03	2.8e+05	7.53e+04	2.8e+06	5.55e+06	1.67e+04	5.18e+04	0.0127	0.0531
-1.35e+03	2.72e+03	2.9e+05	7.16e+04	2.9e+06	5.29e+06	1.65e+04	5.18e+04	0.0134	0.082
-2.72e+03	5.45e+03	3.61e+04	2.45e+04	3.39e+05	3.55e+06	1.81e+04	9.61e+04	0.00218	0.0196
-1.22e+03	2.46e+03	3.04e+05	7.81e+04	3.04e+06	5.38e+06	1.77e+04	5.51e+04	0.0139	0.0688
-1.26e+03	2.53e+03	3.04e+05	7.64e+04	3.04e+06	5.86e+06	1.78e+04	5.5e+04	0.013	0.0623
-1.71e+03	3.44e+03	3.27e+05	7.92e+04	3.27e+06	5.2e+06	1.92e+04	5.03e+04	0.0145	0.00148
-1.74e+03	3.5e+03	2.21e+05	9.07e+04	2.21e+06	5.34e+06	1.89e+04	5.43e+04	0.0128	0.0317
-2.68e+03	5.38e+03	3.4e+04	2.81e+04	2e+05	3.2e+06	1.84e+04	9.06e+04	0.00232	0.0191
-2.72e+03	5.45e+03	3.69e+04	2.3e+04	8.31e+04	3.39e+06	1.76e+04	9.06e+04	0.00233	0.022
-1.25e+03	2.51e+03	2.93e+05	7.76e+04	2.93e+06	5.43e+06	1.7e+04	5.19e+04	0.0138	0.0555
-1.1e+03	2.22e+03	3.09e+05	7.59e+04	3.09e+06	5.76e+06	1.72e+04	5.47e+04	0.0133	0.0786
-1.19e+03	2.39e+03	2.94e+05	7.38e+04	2.94e+06	5.64e+06	1.62e+04	5.22e+04	0.0137	0.0796
-2.81e+03	5.64e+03	1.49e+05	4.99e+04	2.64e+04	3.46e+06	1.62e+04	7.95e+04	0.00791	0.0968
-1.28e+03	2.58e+03	2.95e+05	7.57e+04	2.95e+06	5.31e+06	1.69e+04	5.26e+04	0.0137	0.0771
-1.25e+03	2.51e+03	3e+05	7.56e+04	3e+06	5.6e+06	1.7e+04	5.27e+04	0.0135	0.0696
-1.33e+03	2.67e+03	2.98e+05	8.09e+04	2.98e+06	5.84e+06	1.78e+04	5.51e+04	0.0139	0.0645
-1.03e+03	2.08e+03	2.92e+05	7.14e+04	2.56e+06	5.51e+06	1.62e+04	5.19e+04	0.0134	0.0801

Table S1: Bootstrap parameter estimates for the *D. simulans* and *D. sechellia* joint demographic model obtained via ∂a∂i. The parameters of the model are the ancestral population size (N_{ref}), the final population sizes of *D. sechellia* and *D. simulans* (N_{0-sech} and N_{0-sim}), the initial population sizes (N_{sech} and N_{sim}), the population split time (t_s), and the backwards migration rates ($m_{sim\rightarrow sech}$ and $m_{sech\rightarrow sim}$). Note that parameter estimates are shown for each bootstrap replicate for which our optimization procedure succeeded (Methods), but only those with log-likelihood scores greater than -1750 were used to simulate training data.

bioRxiv preprint doi: https://doi.org/10.1101/2023.02.07.527435; this version posted January 23, 2024. The copyright holder for this preprint (which was not certified by peer review) is the author/funder, who has granted bioRxiv a license to display the preprint in perpetuity. It is made available under aCC-BY 4.0 International license.

Method and problem	Simulation (100k sims)	Formatting	Discriminator training	Segmenter training
`IntroUNET`: simple bidirectional scenario	100.1 h<br>110kb	29 h<br>(2, 64, 128)	0.6 h	4.6 h
`IntroUNET`: Drosophila simulans-sechellia model	4.9 h<br>10kb	21.2 h<br>(2, 32, 128)	0.9 h	3.7 h
`IntroUNET`: Archaic introgression model	10.7 h<br>1Mb	48.5 h<br>(2, 112, 192)	N/A	8.3 h
`ArchIE`: Archaic introgression model	10.7 h	107.4 h<br>50kb	N/A	7 min

Table S2: CPU / GPU time estimates for accomplishing the experiments in the paper. The simulation and formatting results for this table were computed from a small sample of 430 replicates over 4 cores of an Intel Core i9-9900K CPU @ 3.60GHz and then scaled to give estimates for 10^5 replicates in each case. The Formatting column lists the time estimates for alignment sorting (for `IntroUNET`) and statistic calculation (for `ArchIE`). The Discriminator and Segmenter columns list the training times for classifying entire windows and for identifying introgressed haplotypes, respectively. In the GPU columns the estimate is simply the run time for the training described. The training of the neural networks was done on an NVIDIA A40 GPU, and we found that VRAM usage was <12Gb in all cases. We note that the `ArchIE` method computes statistics over the entire simulated window (224.64 on average in our simulated 50kb windows) whereas our method only formats a small sequential sample of SNPs from each replicate (192 for the Archaic introgression program). Below the time estimates for simulation are the simulated region size, and below the formatting times are the resulting "image" size or (populations, individuals, sites) and for the case of `ArchIE`, the window size in base pairs. Note that we do not include the time for execution on data after training, but we observed that classification times for all are generally negligible (although the sorting/statistic calculation steps must be performed first and these can be costly as shown in the Formatting section).
