# Supplementary material for: IntroUNET: identifying introgressed alleles via semantic segmentation
Source: bioRxiv. 2023 Feb 7:2023.02.07.527435. Preprint. [Version 1] doi: 10.1101/2023.02.07.527435 (PMC9979274; doi:10.1101/2023.02.07.527435)
Supplement: Supplementary file 1 [file NIHPP2023.02.07.527435v1-supplement-1.pdf]

bioRxiv preprint doi: https://doi.org/10.1101/2023.02.07.527435; this version posted February 7, 2023. The copyright holder for this preprint (which was not certified by peer review) is the author/funder, who has granted bioRxiv a license to display the preprint in perpetuity. It is made available under aCC-BY 4.0 International license.

7 Supporting Information

bioRxiv preprint doi: https://doi.org/10.1101/2023.02.07.527435; this version posted February 7, 2023. The copyright holder for this preprint (which was not certified by peer review) is the author/funder, who has granted bioRxiv a license to display the preprint in perpetuity. It is made available under aCC-BY 4.0 International license.

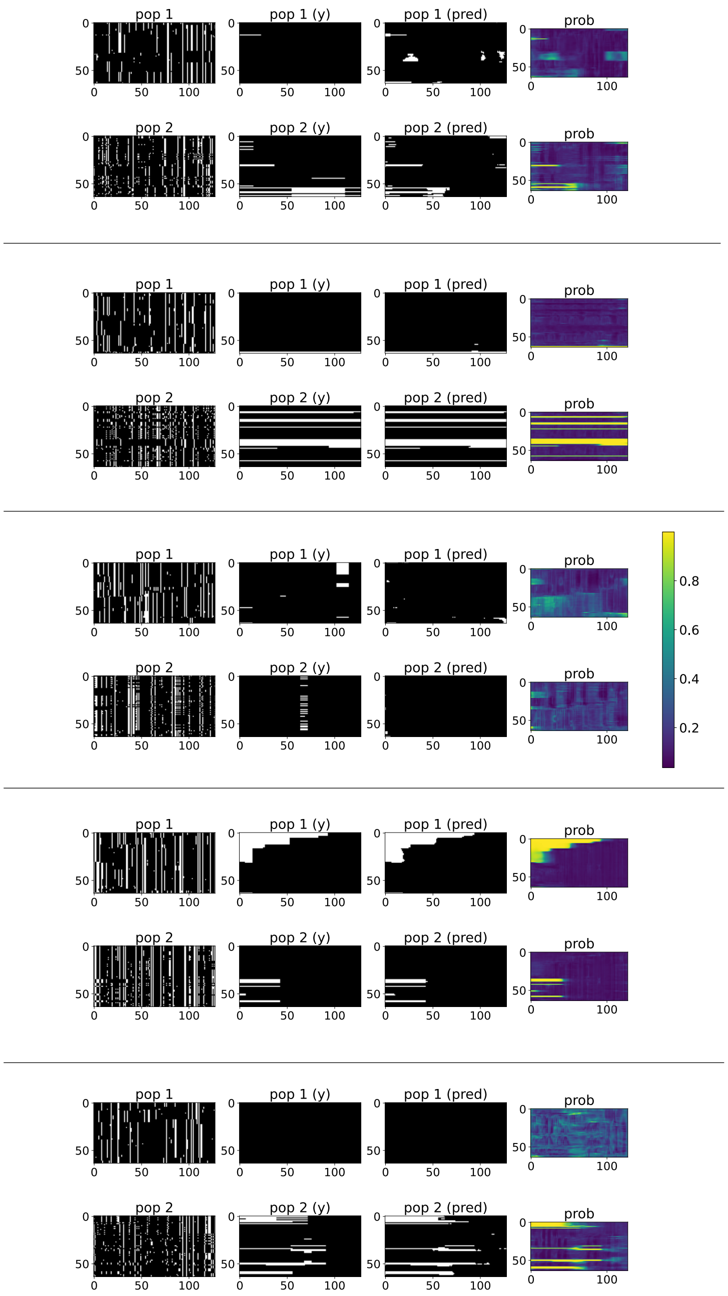

Supplementary Figure 1: Five example segmentations on simulations from our simple bidirectional introgression scenario. Each example shows the input alignments for the two populations (labeled “pop 1” and “pop 2” respectively), the true introgressed alleles for these two populations (labeled “pop 1 (y)” and “pop 2 (y)” respectively), the introgressed alleles inferred by IntroUNET (“pop 1 (pred)” and “pop 2 (pred)”), and IntroUNET’s inferred introgression probabilities (labelled “prob”, and scaled according to the color bar shown with the third example). Alignments and introgressed histories, true and predicted, are shown in the same format as in Figure 1.

bioRxiv preprint doi: https://doi.org/10.1101/2023.02.07.527435; this version posted February 7, 2023. The copyright holder for this preprint (which was not certified by peer review) is the author/funder, who has granted bioRxiv a license to display the preprint in perpetuity. It is made available under aCC-BY 4.0 International license.

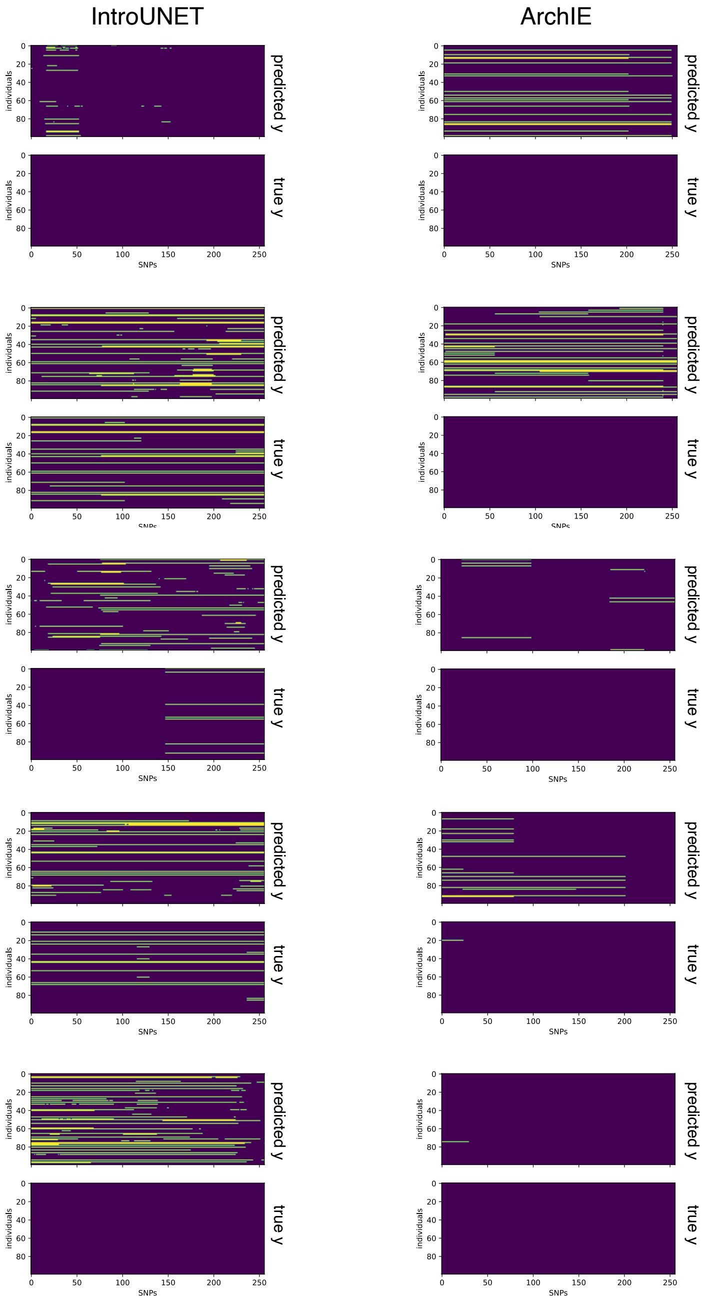

Supplementary Figure 2: Ten example segmentations on simulations from our archaic introgression scenario: 5 from IntroUNET (left) and 5 from ArchIE (right). Each shows the true and inferred introgressed alleles in the recipient population only. For each method, 3 examples with introgression are shown along with 2 examples without introgression.

bioRxiv preprint doi: https://doi.org/10.1101/2023.02.07.527435; this version posted February 7, 2023. The copyright holder for this preprint (which was not certified by peer review) is the author/funder, who has granted bioRxiv a license to display the preprint in perpetuity. It is made available under aCC-BY 4.0 International license.

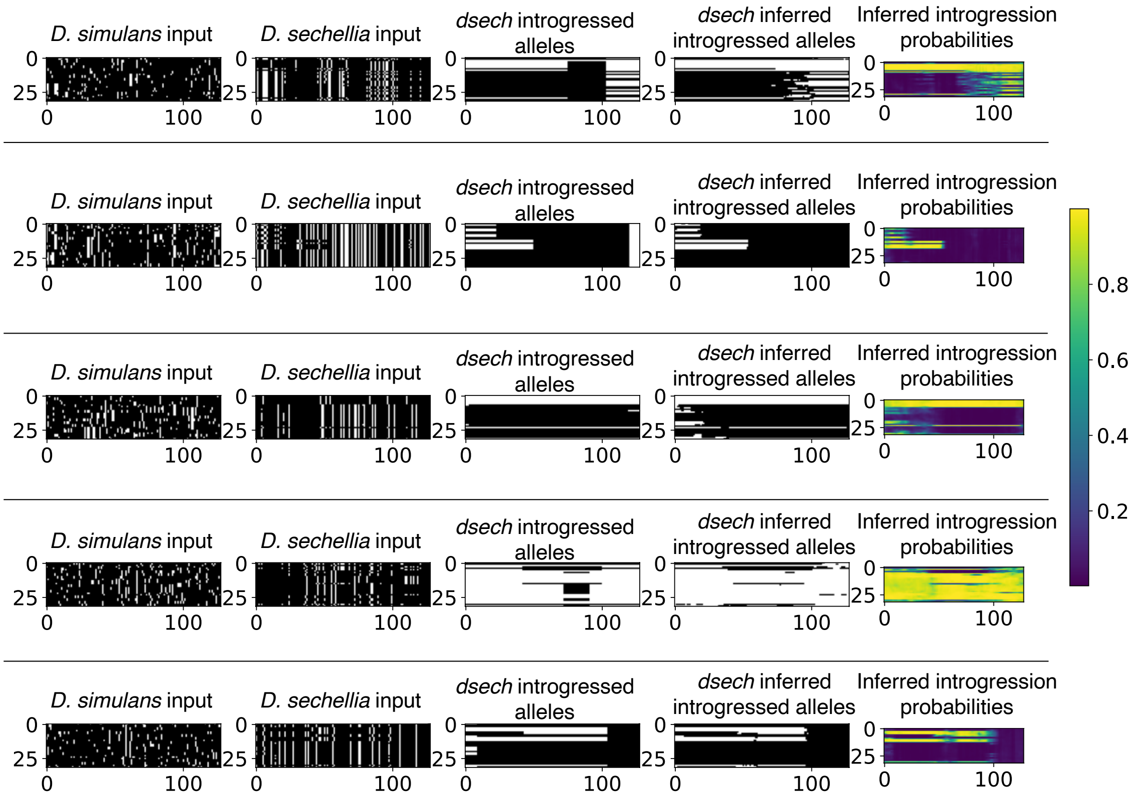

Supplementary Figure 3: Five example segmentations on simulations from our *Drosophila* introgression scenario. Each example shows the input alignments for the two populations, the true and inferred introgressed alleles for the *D. sechellia*population, and IntroUNET's inferred introgression probabilities. Alignments and introgressed histories, true and predicted, are shown in the same format as in Figure 1.

bioRxiv preprint doi: https://doi.org/10.1101/2023.02.07.527435; this version posted February 7, 2023. The copyright holder for this preprint (which was not certified by peer review) is the author/funder, who has granted bioRxiv a license to display the preprint in perpetuity. It is made available under aCC-BY 4.0 International license.

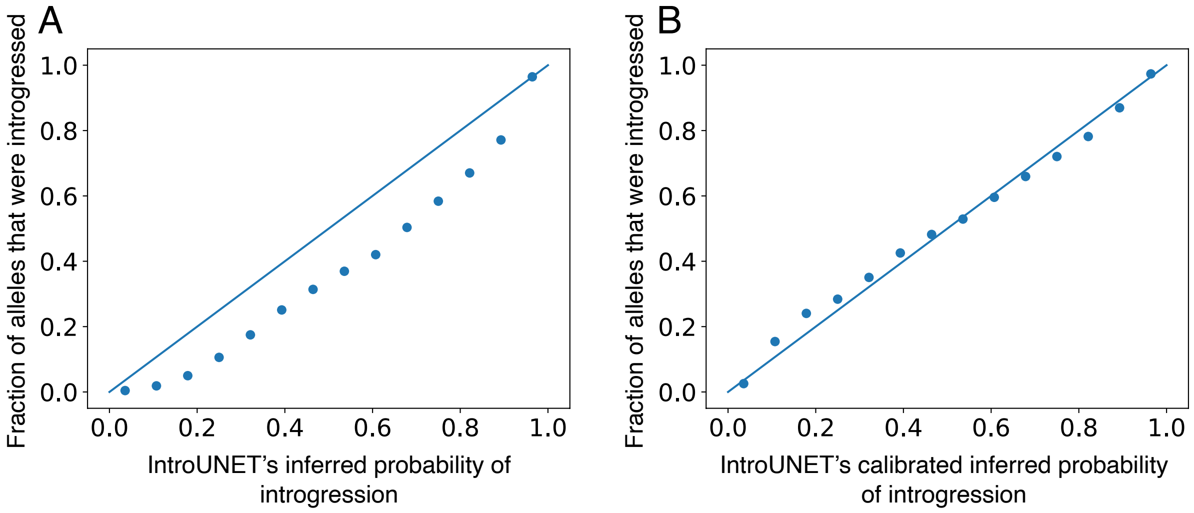

Supplementary Figure 4: Calibration curves showing the impact of Platt scaling on the accuracy of the introgression probability estimates produced by IntroUNET, calculated on the validation set from the *Drosophila* simulations (Methods). A) The fraction of alleles falling within a given bin of IntroUNET's predicted probability of introgression that were in fact truly introgressed, prior to Platt recalibration. B) Same as (A), after recalibration.

bioRxiv preprint doi: https://doi.org/10.1101/2023.02.07.527435; this version posted February 7, 2023. The copyright holder for this preprint (which was not certified by peer review) is the author/funder, who has granted bioRxiv a license to display the preprint in perpetuity. It is made available under aCC-BY 4.0 International license.

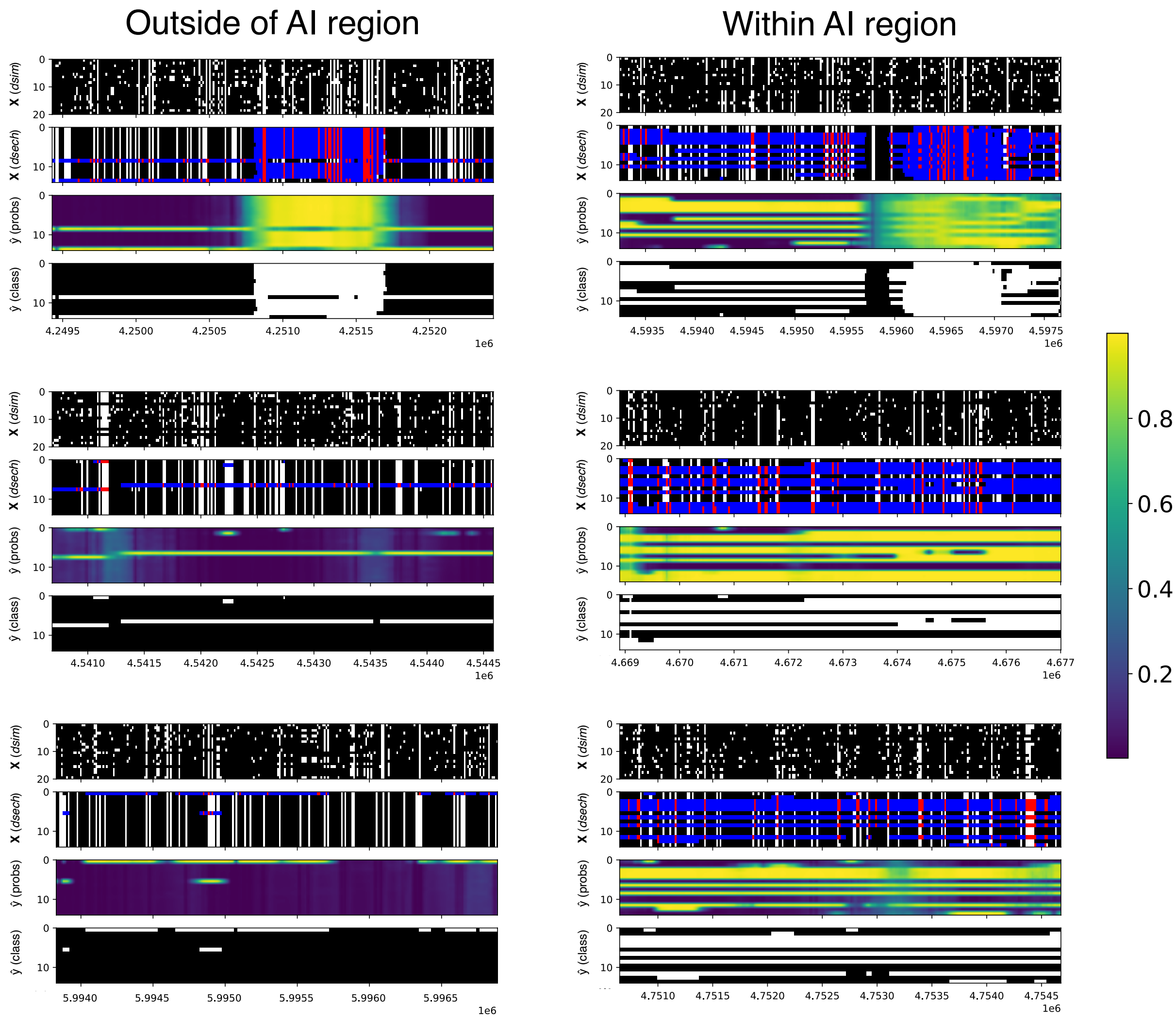

Supplementary Figure 5: Alignments and segmentations from six windows on chr3R in the vicinity of the locus of adaptive introgression (AI). Three of the windows (left) are from outside of the region affected by AI, and the other three (right) are within the AI locus. Each example shows the input alignments for the two populations (labeled "**X** (*dsim*)" and "**X** (*dsech*)", respectively), IntroUNET's inferred introgression probabilities ("$\hat{y}$ (probs)"), and the most probable class for each allele in each individual ("$\hat{y}$ (class)"). Inferred introgressed histories and introgression probabilities are shown in the same format as in Figure 1. Alignments are shown in the same format as for previous figures, with the exception that for *D. sechellia* which has a different color scheme in haplotypes that were inferred to be introgressed in order to highlight these regions of the alignment: blue for the ancestral allele, and red for the derived allele.

bioRxiv preprint doi: https://doi.org/10.1101/2023.02.07.527435; this version posted February 7, 2023. The copyright holder for this preprint (which was not certified by peer review) is the author/funder, who has granted bioRxiv a license to display the preprint in perpetuity. It is made available under aCC-BY 4.0 International license.

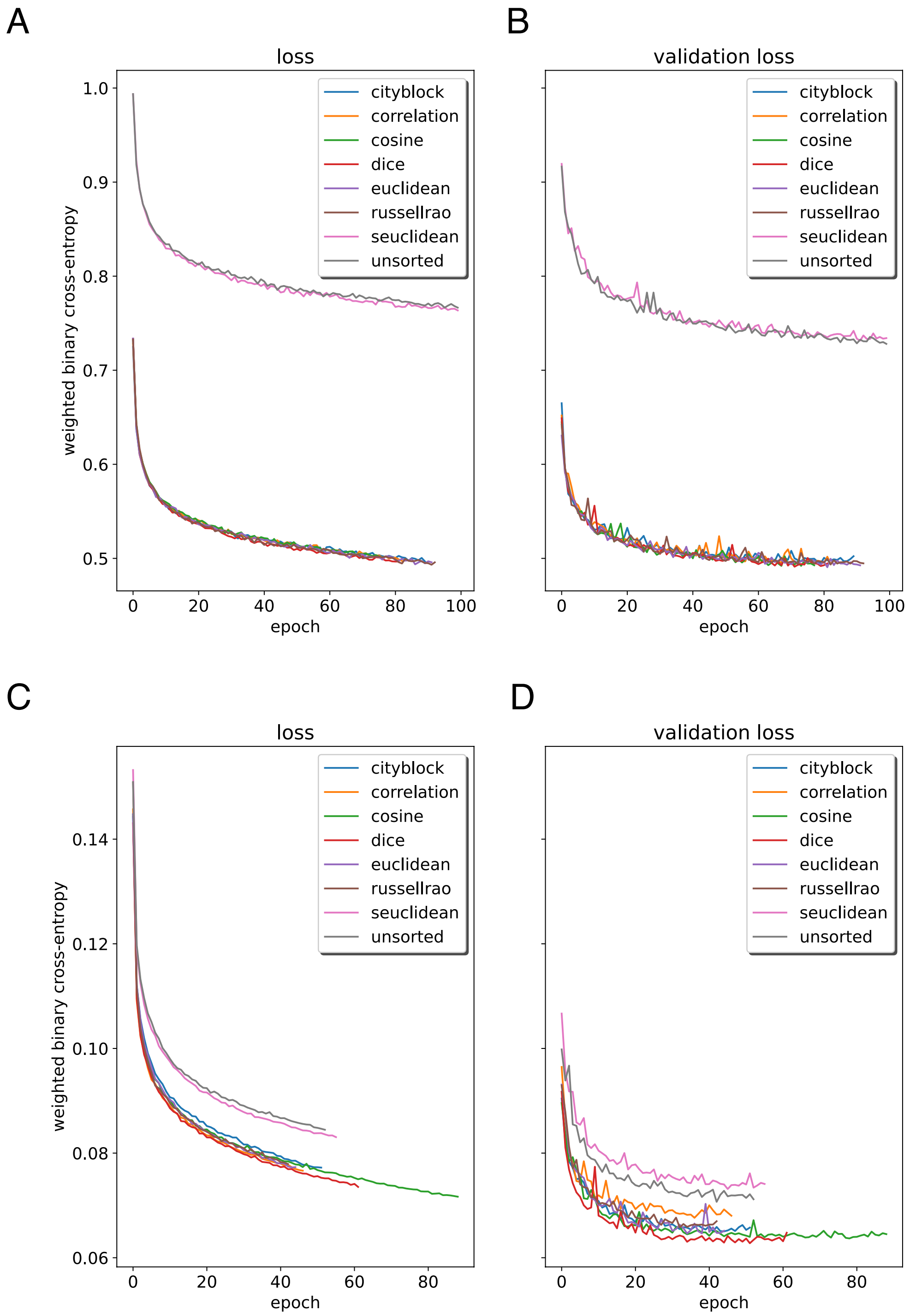

Supplementary Figure 6: Results of training the same architecture on data seriated via different distance metrics, as well as using unsorted data (i.e. individuals within each population are arranged in the arbitrary order produced by the simulator), for the ghost-introgression problem (top row, panels A and B) and the *Drosophila* demographic model (bottom row, panels C and D). These plots show the values of training (A and C) and validation (B and D) loss over the course of training. Validation loss is usually lower than training in the case of *Drosophila* because label smoothing was applied to the training data for the purposes of regularization, but not to the validation data.

bioRxiv preprint doi: https://doi.org/10.1101/2023.02.07.527435; this version posted February 7, 2023. The copyright holder for this preprint (which was not certified by peer review) is the author/funder, who has granted bioRxiv a license to display the preprint in perpetuity. It is made available under aCC-BY 4.0 International license.

Log-likelihood	AIC	N	α_0	α_1	ν_0	ν_1	T	m_{01}	m_{10}
-1.54e+03	3.1e+03	3.27e+05	7.47e+04	3.27e+06	5.94e+06	1.55e+04	5.31e+04	0.0104	0.141
-1.42e+03	2.85e+03	2.86e+05	7.29e+04	2.86e+06	5.55e+06	1.72e+04	5.11e+04	0.0136	0.061
-1.27e+03	2.56e+03	2.94e+05	7.82e+04	2.94e+06	5.79e+06	1.75e+04	5.4e+04	0.0138	0.05
-1.28e+03	2.58e+03	3.04e+05	7.81e+04	3.04e+06	5.46e+06	1.79e+04	5.36e+04	0.0143	0.0648
-1.26e+03	2.54e+03	2.88e+05	7.42e+04	2.88e+06	5.14e+06	1.73e+04	5.12e+04	0.0133	0.0595
-1.27e+03	2.55e+03	3.12e+05	8.1e+04	3.12e+06	5.7e+06	1.8e+04	5.53e+04	0.0141	0.0694
-2.99e+03	5.99e+03	3.56e+04	2.54e+04	2.02e+05	3.43e+06	1.84e+04	9.49e+04	0.00216	0.0204
-1.15e+03	2.32e+03	2.91e+05	7.67e+04	2.91e+06	5.16e+06	1.65e+04	5.24e+04	0.0129	0.0528
-1.07e+03	2.15e+03	2.84e+05	7.16e+04	2.84e+06	5.16e+06	1.62e+04	5.06e+04	0.0132	0.0589
-1.64e+03	3.29e+03	2.91e+05	7.25e+04	2.93e+05	5.15e+06	2.06e+04	5.51e+04	0.0116	0.103
-1.4e+03	2.8e+03	3.04e+05	7.6e+04	3.04e+06	5.32e+06	1.72e+04	5.31e+04	0.0136	0.0734
-2.98e+03	5.97e+03	3.64e+04	2.06e+04	2.25e+05	3.48e+06	1.77e+04	9.71e+04	0.00216	0.0239
-1.2e+03	2.41e+03	2.91e+05	7.23e+04	2.9e+06	5.05e+06	1.66e+04	5.09e+04	0.0132	0.078
-1.11e+03	2.23e+03	3.04e+05	8.15e+04	3.04e+06	5.67e+06	1.73e+04	5.62e+04	0.0137	0.0675
-1.25e+03	2.51e+03	2.94e+05	7.71e+04	2.94e+06	5.5e+06	1.72e+04	5.38e+04	0.013	0.0571
-1.31e+03	2.64e+03	2.95e+05	7.98e+04	2.95e+06	5.42e+06	1.69e+04	5.34e+04	0.0134	0.0529
-1.14e+03	2.29e+03	2.73e+05	6.64e+04	2.73e+06	5.14e+06	1.56e+04	4.78e+04	0.0131	0.0573
-1.23e+03	2.48e+03	2.95e+05	7.16e+04	2.95e+06	5.44e+06	1.72e+04	5.06e+04	0.0137	0.0626
-3.13e+03	6.27e+03	3.62e+04	2.42e+04	2.84e+05	3.46e+06	1.79e+04	9.44e+04	0.00223	0.0225
-2.96e+03	5.94e+03	4.24e+04	2.53e+04	2.99e+05	3.58e+06	1.84e+04	1.01e+05	0.00247	0.0255
-2.89e+03	5.79e+03	3.95e+04	2.42e+04	6.15e+04	3.6e+06	1.79e+04	9.48e+04	0.0024	0.0213
-1.24e+03	2.49e+03	2.94e+05	7.79e+04	2.94e+06	5.82e+06	1.66e+04	5.38e+04	0.013	0.0662
-1.43e+03	2.88e+03	2.98e+05	7.5e+04	2.98e+06	5.47e+06	1.77e+04	5.17e+04	0.0144	0.0688
-1.29e+03	2.6e+03	3.08e+05	7.78e+04	3.08e+06	5.69e+06	1.74e+04	5.55e+04	0.0132	0.0848
-1.33e+03	2.66e+03	3.01e+05	7.75e+04	3.01e+06	5.66e+06	1.74e+04	5.3e+04	0.0137	0.0567
-1.27e+03	2.56e+03	2.87e+05	7.73e+04	2.87e+06	5.05e+06	1.66e+04	5.19e+04	0.0133	0.0649
-2.88e+03	5.78e+03	3.87e+04	2.57e+04	3.87e+05	3.44e+06	1.86e+04	9.52e+04	0.00236	0.0231
-1.31e+03	2.62e+03	3e+05	7.83e+04	3e+06	5.68e+06	1.71e+04	5.43e+04	0.0136	0.0767
-1.26e+03	2.53e+03	3.05e+05	8.19e+04	3.05e+06	5.85e+06	1.78e+04	5.47e+04	0.0135	0.0542
-1.34e+03	2.69e+03	3.01e+05	7.67e+04	3.01e+06	5.65e+06	1.75e+04	5.31e+04	0.0134	0.0666
-1.35e+03	2.72e+03	2.94e+05	7.76e+04	2.94e+06	5.47e+06	1.72e+04	5.29e+04	0.0131	0.0654
-2.68e+03	5.38e+03	3.72e+04	2.5e+04	3.72e+05	3.72e+06	1.91e+04	9.91e+04	0.00239	0.0189
-1.28e+03	2.57e+03	2.81e+05	7.61e+04	2.81e+06	5.43e+06	1.66e+04	5.15e+04	0.0134	0.0642
-2.84e+03	5.69e+03	3.66e+04	2.58e+04	3.11e+05	3.52e+06	1.79e+04	9.72e+04	0.00221	0.0195
-1.18e+03	2.36e+03	2.93e+05	7.66e+04	2.93e+06	5.32e+06	1.65e+04	5.23e+04	0.0134	0.0628
-1.26e+03	2.54e+03	2.9e+05	7.13e+04	2.9e+06	5.42e+06	1.65e+04	5.01e+04	0.0133	0.0538
-3e+03	6.01e+03	4.94e+04	2.75e+04	4.94e+05	3.29e+06	1.74e+04	9.03e+04	0.00318	0.028
-1.24e+03	2.49e+03	2.92e+05	7.67e+04	2.92e+06	5.66e+06	1.74e+04	5.24e+04	0.0139	0.0415
-1.35e+03	2.71e+03	3.04e+05	7.74e+04	3.04e+06	5.33e+06	1.7e+04	5.32e+04	0.0138	0.0694
-2.51e+03	5.04e+03	3.57e+04	2.37e+04	1.51e+05	3.47e+06	1.66e+04	9.52e+04	0.00231	0.0225
-1.23e+03	2.48e+03	2.8e+05	7.53e+04	2.8e+06	5.55e+06	1.67e+04	5.18e+04	0.0127	0.0531
-1.35e+03	2.72e+03	2.9e+05	7.16e+04	2.9e+06	5.29e+06	1.65e+04	5.18e+04	0.0134	0.082
-2.72e+03	5.45e+03	3.61e+04	2.45e+04	3.39e+05	3.55e+06	1.81e+04	9.61e+04	0.00218	0.0196
-1.22e+03	2.46e+03	3.04e+05	7.81e+04	3.04e+06	5.38e+06	1.77e+04	5.51e+04	0.0139	0.0688
-1.26e+03	2.53e+03	3.04e+05	7.64e+04	3.04e+06	5.86e+06	1.78e+04	5.5e+04	0.013	0.0623
-1.71e+03	3.44e+03	3.27e+05	7.92e+04	3.27e+06	5.2e+06	1.92e+04	5.03e+04	0.0145	0.00148
-1.74e+03	3.5e+03	2.21e+05	9.07e+04	2.21e+06	5.34e+06	1.89e+04	5.43e+04	0.0128	0.0317
-2.68e+03	5.38e+03	3.4e+04	2.81e+04	2e+05	3.2e+06	1.84e+04	9.06e+04	0.00232	0.0191
-2.72e+03	5.45e+03	3.69e+04	2.3e+04	8.31e+04	3.39e+06	1.76e+04	9.06e+04	0.00233	0.022
-1.25e+03	2.51e+03	2.93e+05	7.76e+04	2.93e+06	5.43e+06	1.7e+04	5.19e+04	0.0138	0.0555
-1.1e+03	2.22e+03	3.09e+05	7.59e+04	3.09e+06	5.76e+06	1.72e+04	5.47e+04	0.0133	0.0786
-1.19e+03	2.39e+03	2.94e+05	7.38e+04	2.94e+06	5.64e+06	1.62e+04	5.22e+04	0.0137	0.0796
-2.81e+03	5.64e+03	1.49e+05	4.99e+04	2.64e+04	3.46e+06	1.62e+04	7.95e+04	0.00791	0.0968
-1.28e+03	2.58e+03	2.95e+05	7.57e+04	2.95e+06	5.31e+06	1.69e+04	5.26e+04	0.0137	0.0771
-1.25e+03	2.51e+03	3e+05	7.56e+04	3e+06	5.6e+06	1.7e+04	5.27e+04	0.0135	0.0696
-1.33e+03	2.67e+03	2.98e+05	8.09e+04	2.98e+06	5.84e+06	1.78e+04	5.51e+04	0.0139	0.0645
-1.03e+03	2.08e+03	2.92e+05	7.14e+04	2.56e+06	5.51e+06	1.62e+04	5.19e+04	0.0134	0.0801

Supplementary Table 1: Bootstrap parameter estimates for the *D. simulans* and *D. sechellia* joint demographic model obtained via ∂a∂i.
